# Predictors, barriers and facilitators of bystander interventions in out of hospital cardiac arrest: a cross-sectional study from the UAE

**DOI:** 10.3389/fpubh.2026.1738145

**Published:** 2026-02-23

**Authors:** Uffaira Hafeez, Azhar T. Rahma, Aminu S. Abdullahi, Messaouda Belfakir, Khalifa Alseiari, Mohammad Ali Alsaadi, Nasser Abdulla Alshamsi, Omar Alzaabi, Saoud Al Tamimi, Khalid Almaamari, Munawar Farooq

**Affiliations:** 1 Department of Internal Medicine, Emergency Medicine Section, College of Medicine and Health Sciences, United Arab Emirates University (UAEU), Al Ain, United Arab Emirates; 2 Institute of Public Health, College of Medicine and Health Sciences, UAEU, Al Ain, United Arab Emirates; 3 College of Medicine and Health Sciences, United Arab Emirates University (UAEU), Al Ain, United Arab Emirates; 4 Department of Emergency Medicine, Tawam Hospital, Al Ain, United Arab Emirates

**Keywords:** AED, bystander, cardiopulmonary resuscitation, community willingness, defibrillation, out of hospital cardiac arrest

## Abstract

**Introduction:**

Out-of-Hospital Cardiac Arrest (OHCA) has low survival rates, especially in the Middle East, where bystander response in the UAE remains limited. Early cardiopulmonary resuscitation (CPR) and automated external defibrillator (AED) use improve outcomes. This study assessed public willingness to intervene during OHCA and identified key predictors, barriers, and facilitators.

**Methods:**

A cross-sectional survey of 1,020 UAE adults (18+) was conducted using a 35-item, expert-validated questionnaire. Participants were recruited via convenience and snowball sampling, both online and in person. Descriptive statistics and binary logistic regression were conducted to identify factors associated with willingness to intervene.

**Results:**

Responses were predominantly from younger, educated individuals, with South Asians underrepresented relative to the overall UAE population. CPR and AED training were reported by 52 and 34% of participants, respectively. Training was lower among females, South Asians, and those with lower educational attainment. Training correlated with greater confidence and positive attitudes; however, both trained and untrained individuals reported similar cultural and legal barriers to bystander response. Approximately 60.6% of survey responders were willing to perform CPR, while 46.8% were willing to use an AED. Women were more likely to perform CPR on young females, while men were more likely to assist young males. Prior training emerged as the strongest predictor of willingness, with repeated training further increasing willingness alongside being a healthcare provider, confidence in recognizing cardiac arrest, and positive attitudes toward bystander intervention. The main barriers included a lack of skill, legal concerns, and low confidence, while key facilitators were dispatcher guidance and legal protection. Among trained but unwilling participants, more than half cited low confidence as a barrier to performing CPR and using an AED.

**Conclusion:**

Addressing observed demographic, regional, and cultural disparities in training and willingness through targeted public health strategies could support improved bystander response in the UAE.

## 1 Introduction

Out-of-Hospital Cardiac Arrest (OHCA) is a major public health challenge. The true burden of OHCA remains uncertain due to underreporting and the regional differences in data collection (1). Global data compiled by the International Liaison Committee on Resuscitation from 15 registries across North America, Europe, Asia, and Oceania estimated the incidence of EMS-treated OHCA at 30.0–100.2 per 100,000 population between 2015 and 2017, with survival to hospital discharge or 30-day survival ranging from 4.6 to 16.4%, underscoring substantial international disparities in outcomes (2). Official data sources and comprehensive population-based studies remain limited in the Middle East. Available evidence is largely derived from individual center-based studies, which report high mortality rates across several Gulf Cooperation Council countries (3). Overall, survival rates in the Middle East and Asia appear lower than global averages, with most regional studies reporting survival rates below 10% (4, 5). The PAROS study, which included data from Dubai, reported a survival rate of 3% in the United Arab Emirates (UAE) (6).

Bystander response plays a crucial role in the “Chain of Survival” framework, which emphasizes early recognition of cardiac arrest, Emergency Medical Services (EMS) activation, bystander cardiopulmonary resuscitation (CPR), and early defibrillation (7). The EuReCa TWO study across 28 European countries reported a 9.1% survival rate when CPR was initiated by bystanders, compared to 4.5% when started by EMS (8). CPR can increase survival two- to threefold (9, 10), while public automated external defibrillator (AED) use may raise survival chances by up to 75% (11). Despite this, bystander CPR rates range widely from 13% in Serbia to 82% in Norway (8), and AED use varies from 9.3% in Denmark to 59.3% in the Netherlands (12, 13). Various global initiatives have been introduced to improve both CPR and AED use (14, 15).

Data on OHCA outcomes in GCC countries is limited, with lower survival rates, decreased bystander response rates, and lower rates of AED use (5, 16). The PAROS study indicated a bystander response rate of 10.5% in the UAE, the lowest among the seven countries included from Asia and a bystander AED use rate of 0.8% (6). According to the Theory of Planned Behavior, an individual’s intention to act is influenced by attitudes, perceived social norms, and perceived control (17). While various studies from other regions have examined public attitudes and willingness to intervene in OHCA, there is a literature gap in the Middle East regarding attitudes, sociocultural and demographic factors influencing bystander response. The objective of this study was to identify predictors of willingness to perform CPR and use an AED, and to describe barriers and facilitators to bystander CPR and AED use in the UAE.

## 2 Materials and methods

### 2.1 Study design

A cross-sectional survey design was employed.

### 2.2 Questionnaire

A 35-item survey was developed based on a literature review and validated for content and face validity by experts in public health and emergency medicine. It was translated into Arabic and Urdu by native speakers and back-translated for accuracy. The final versions were agreed upon with consensus within the research team (Supplementary material).

### 2.3 Study participants

Adults aged 18 years and older who were either nationals or residents of the UAE were eligible to participate.

### 2.4 Sample size

The sample size for the present study was statistically estimated using Epi-Info software, based on the sample size calculation formula for cross-sectional (prevalence) studies: 𝑛 = 𝑍^2^p(1 − p)/𝑑^2^ (18). Where n is the required optimal sample size, Z is the Z-score for 95% confidence interval corresponding to 1.96, p is the expected proportion of bystanders responding to OHCA, and d is the margin of error, set at 2% to achieve high precision. For the expected proportion of bystanders responding to OHCA, 10.5% was used, as reported in PAROS study (6). This yielded a sample size of 902 participants. Accounting for an anticipated 10% non-response rate, the target sample was increased to 992. In total, 1,021 survey submissions were received.

### 2.5 Data collection

Data collection was conducted between July and November 2024 through both online and in-person approaches. Convenience sampling and the snowball technique were employed to increase participation. The survey was disseminated via official university mailing lists and social media platforms (e.g., WhatsApp, LinkedIn, Facebook). The SurveyMonkey platform was used for online responses. Medical students administered the survey in public venues (e.g., shopping malls, parks and community centers) using tablets. The CHERRIES (Checklist for Reporting Results of Internet E-Surveys) is provided in Supplementary material (19).

### 2.6 Data analysis

Categorical variables were summarized as frequencies and percentages, while age, as a continuous variable, was expressed as the mean and standard deviation. Associations between categorical variables were evaluated using chi-squared tests. The outcomes, willingness to perform CPR and willingness to use an AED, were coded as binary variables. Specifically, participants who responded “definitely yes” or “probably yes” were classified as willing (coded 1), whereas those who responded “definitely no” or “probably no” were classified as unwilling (coded 0). Binary logistic regression models were constructed to identify predictors of willingness to provide CPR and use an AED. Missing data were minimal (<7% across variables), with no missing data in the outcome variables therefore complete case analysis was applied using using R’s glm() function.

For multivariable analysis, variables with *p*-values < 0.1 in the univariate models (Appendix Table A5) were included in the multivariable model to retain potentially relevant predictors while minimizing overfitting. Statistical significance was set at *p* < 0.05. The assumption of independence was ensured, as each response was collected from a unique participant, with one response permitted per browser. For in-person tablet-based data collection, surveys were administered individually by data collectors, ensuring that each participant completed the survey only once. Multicollinearity was assessed using variance inflation factors (VIF), with all values remaining below our conservative threshold of 5, indicating no concerning collinearity among predictors (20).

### 2.7 Ethical considerations

The study was approved by the UAE University Ethics Board (ERSC_2024_4460). All participants provided informed consent.

## 3 Results

The study included 1,020 participants (mean age 28 ± 11 years), mostly young adults aged 18–35 (76%), with 60% women and 71% being Emiratis. Nearly half of the participants (49%) lived in Al-Ain, and 22% were healthcare providers. Only 51% knew the correct emergency contact number (Table 1).

**Table 1:** Demographics, characteristics and training status of participants (*N* = 1,020).

Study variables	Categories	*N* (%)
Demographics		
Age	18–35	771 (76%)
	36–49	192 (19%)
	50 and above	57 (5%)
Sex	Female	611 (60%)
Marital status	Married	325 (32%)
	Unmarried	656 (64%)
	Divorced/widowed	39 (3.8%)
Location	Abu Dhabi	326 (32%)
	Al Ain	501 (49%)
	Dubai	75 (7.4%)
	Others (Sharjah, Fujairah, Ras al Khaima, Ajman, Umm Al-Quwain)	118 (12%)
Ethnicity	Emirati	727 (71%)
	Other Arab	152 (15%)
	South Asian	92 (9.0%)
	Others	49 (5%)
Education level	Primary/secondary school	212 (21%)
	College/University	599 (59%)
	Postgraduate	201 (20%)
Employment	Employed	669 (66%)
	Unemployed	351 (34%)
Monthly income (Emirati Dirhams)	< 15,000	274 (50%)
	15,000-29,9,999	133 (24%)
	30,000-44,999	87 (16%)
	> = 45,000	55 (10%)
Characteristics		
Healthcare providers		227 (22%)
Know correct medical emergency number		493(51%)
Personal history of heart disease		70 (7.1%)
Family history of heart disease		365 (37%)
Residing with family member aged >65		442 (45%)
Believe that bystander CPR can increase survival (positive attitude)		888 (94%)
Confident in recognizing cardiac arrest		436 (45%)
Witnessed arrest		165 (21%)
Training status		
Trained for CPR		459 (52%)
Frequency of training among trained (CPR) (*n* = 459)	1 time	159 (38%)
	2 times	104 (24%)
	3 times or more	159 (38%)
Last Trained (CPR) (*n* = 459)	Less than 1 year ago,	204 (48%)
	More than 1 year ago but within 5 years	151 (36%)
	More Than 5 years ago	69 (16%)
Trained for AED		278 (34%)
Frequency of training among trained (AED) (*n* = 278)	1 time	77 (33%)
	2 times	65 (28%)
	3 times or more	92 (39%)
Last Trained (AED) (*n* = 278)	Less than 1 year ago,	152 (63%)
	More than 1 year ago but within 5 years	76 (32%)
	More Than 5 years ago	12 (5.0%)

*Percentages are calculated excluding non-responses and some variables may not sum to 100%.

### 3.1 Training

Overall, 52% of participants had received CPR training, and 34% had received AED training, with rates varying significantly by sex, education, location, and nationality (Appendix Tables A1–A2). Males were more likely than females to be trained in CPR (58% vs. 48%, *p* = 0.003). Ethnicity was significantly associated with both CPR and AED training (*p* < 0.001) with South Asians reporting lowest rates (CPR 22% vs. 56%; AED 11% vs. 37% in Emiratis *p* < 0.001). Education level influenced AED training, increasing from 28% among those with primary/secondary education to 45% among postgraduates (*p* = 0.003). Both personal and family history of heart disease were linked to higher training rates (CPR: 74% vs. 50 and 59% vs. 48%; AED: 69% vs. 31 and 41% vs. 30%; all *p* < 0.001). Belief in bystander CPR effectiveness (54% vs. 19%) and witnessing a cardiac arrest (CPR 78% vs. 47%; AED 71% vs. 24%, all *p* < 0.001) were strongly associated with training. Confidence showed a dose-dependent association for both (*p* < 0.001) with training rising from 15% CPR/4% AED among the “Not confident” to 82% CPR/85% AED among the “Very confident” (Appendix Tables A1, A2).

### 3.2 Willingness to perform CPR and use an AED

On bivariate analysis, willingness to perform CPR and use an AED was associated with higher education (CPR: 68% in postgraduates vs. 57% in primary/secondary, *p* = 0.044; AED: 56% vs. 38%, *p* < 0.001), healthcare provider status (CPR:80% vs. 55%; AED:70% vs. 40%, *p* < 0.001) and location, with respondents from the Northern Emirates less willing than those from Abu Dhabi (CPR: 44% vs. 67%, *p* < 0.001; AED: 36% vs. 51%, *p* = 0.048). Attitudinal and experiential factors including positive attitude (CPR: 67% vs. 46%, *p* = 0.002; AED: 52% vs. 26%, *p* < 0.001), recognition of cardiac arrest (CPR: 77% vs. 54%; AED: 60% vs. 39%, p < 0.001), and witnessing an arrest (CPR: 87% vs. 66%; AED: 78% vs. 53%, *p* < 0.001) also predicted greater willingness, as did history of heart disease (CPR: 74% vs. 61%, *p* = 0.033; AED: 70% vs. 47%, *p* < 0.001). Training showed the strongest dose–response effects: prior training (CPR: 84% vs. 56%; AED: 88% vs. 43%, *p* < 0.001) repeated sessions (CPR: 94% vs. 69%, *p* < 0.001; AED: 91% vs. 81%, *p* = 0.045) and more recent exposure (CPR: 89% vs. 68%, *p* < 0.001; AED: 91% vs. 84%, *p* = 0.005) were all linked to higher willingness. Confidence in performing CPR demonstrated a clear gradient ranging from 41% (CPR) and 29% (AED) among participants who reported “not confident” to 93% (CPR) and 92% (AED) among those who reported “very confident” (*p* < 0.001 for both) (Appendix Tables A3, A4).

In the multivariable analysis, prior training emerged as the strongest and most consistent predictor of willingness for both CPR and AED use, demonstrating clear dose-dependent effects. For CPR, training once (OR 1.65, 95% CI 1.02–2.69, *p* = 0.041), twice (OR 4.12, 95% CI 2.13–8.57, *p* < 0.001), and three or more times (OR 6.73, 95% CI 3.07–17.0, *p* < 0.001) was associated with progressively higher willingness. Similarly, for AED use, training once (OR 5.10, 95% CI 2.00–14.5, *p* = 0.001), twice (OR 27.1, 95% CI 7.29–178, *p* < 0.001), and three or more times (OR 22.1, 95% CI 7.19–85.8, *p* < 0.001) substantially increased willingness.

For CPR, willingness was independently higher among healthcare providers (OR 2.33, 95% CI 1.13–5.21, *p* = 0.029) and those confident in recognizing cardiac arrest (OR 1.58, 95% CI 1.03–2.43, *p* = 0.036), whereas for AED use, belief that bystander CPR can improve survival independently predicted willingness (OR 3.90, 95% CI 1.14–18.3, *p* = 0.048) (Table 2).

**Table 2:** Odds ratios from multivariable logistic regression for willingness to perform CPR and Use AED.

Characteristics	Categories	CPR Willingness		AED Willingness	
		OR (95% CI)	*p*-value	OR (95% CI)	*p*-value
Sex (ref: Female)	Male	0.93 (0.61, 1.44)	0.80	0.86 (0.51, 1.46)	0.60
Marital status (ref: Married)	Unmarried	1.03 (0.55, 1.93)	>0.90	1.13 (0.65, 1.97)	0.7
	Divorced/widowed	0.53 (0.17, 1.67)	0.30	0.48 (0.17, 1.33)	
Education level (ref: Primary/secondary school)	College/University (bachelor’s degree or equivalent)	1.02 (0.65, 1.60)	>0.90	1.31 (0.62, 2.82)	0.5
	Postgraduate	1.12 (0.52, 2.46)	0.80	1.78 (0.79, 4.06)	0.20
Employment (ref: Employed)	Student	0.78 (0.44, 1.38)	0.40	–	–
	Unemployed	1.31 (0.56, 3.23)	0.50	–	–
Income level (ref: < 30,000 AED)	>30,000 AED	–		0.89 (0.45, 1.73)	0.7
Location (ref: Abu Dhabi)	Al ain	0.84 (0.54, 1.31)	0.50	1.63 (0.92, 2.93)	0.10
	Dubai	0.75 (0.36, 1.59)	0.40	1.70 (0.60, 4.93)	0.3
	Northern Emirates (Sharjah, Fujairah, Ras al Khaima, Ajman, Umm Al-Quwain)	0.70 (0.38, 1.32)	0.30	2.02 (0.73, 5.73)	0.2
Ethnicity (ref: Emiratis)	Other Arabs	–		0.56 (0.28, 1.10)	0.095
	South Asians	–		0.51 (0.24, 1.09)	0.084
	Others	–		0.30 (0.10, 0.81)	**0.020**
Healthcare Provider (ref: No)	Yes	2.33 (1.13, 5.21)	**0.029**	1.08 (0.54, 2.17)	0.80
Trained (ref: Not trained)	Trained once	1.65 (1.02, 2.69)	**0.041**	5.10 (2.00, 14.5)	**0.001**
	Trained twice	4.12 (2.13, 8.57)	**<0.001**	27.1 (7.29, 178)	**<0.001**
	Trained thrice or more	6.73 (3.07, 17.0)	**<0.001**	22.1 (7.19, 85.8)	**<0.001**
Positive attitude towards CPR (ref: No)	Yes	1.15 (0.53, 2.51)	0.70	3.90 (1.14, 18.3)	**0.048**
History of heart Disease (ref: No)	Yes	1.24 (0.43, 4.06)	0.70	1.62 (0.51, 5.29)	0.40
Family history of heart disease (ref: No)	Yes	0.79 (0.52, 1.21)	0.30	1.23 (0.73, 2.08)	0.4
Residing with family member aged >65 (ref: No)	Yes	–	–	1.51 (0.89, 2.55)	0.13
Ability to recognize cardiac arrest	Yes	1.58 (1.03, 2.43)	**0.036**	0.95 (0.54, 1.64)	0.8
Witnessed cardiac arrest (ref: No)	Yes	1.57 (0.85, 3.04)	0.2	1.33 (0.61, 2.87)	0.5

Variables with *p* > 0.1 in the univariable analysis were excluded from the multivariable model and are represented as “—”.

**Figure 1:**
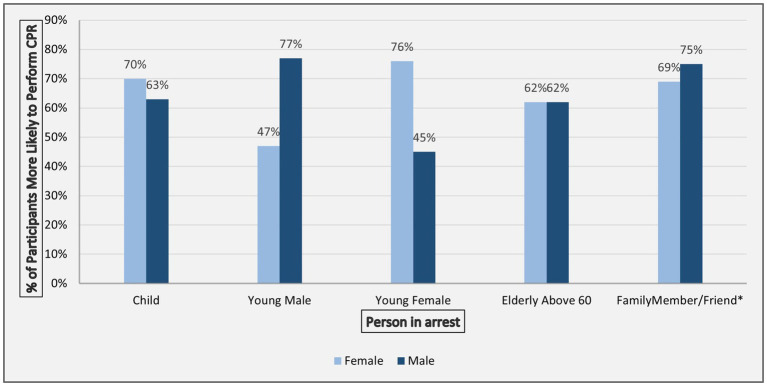
Proportion of male and female respondents more likely to perform CPR based on different individuals in cardiac arrest.

The likelihood of performing CPR varied significantly based on the sex of the individual in cardiac arrest. Women were more likely than men to perform CPR on children (70% vs. 63%, *p* = 0.024) and young females (76% vs. 45%, *p* < 0.001), whereas men were more likely to perform CPR on young males (77% vs. 47%, *p* < 0.001) (Figure 1). For AED use, the difference between men and women when intervening with male individuals was not statistically significant (61% vs. 55%, *p* = 0.09)

### 3.3 Barriers and facilitators to performing CPR and using an AED

**Figure 2:**
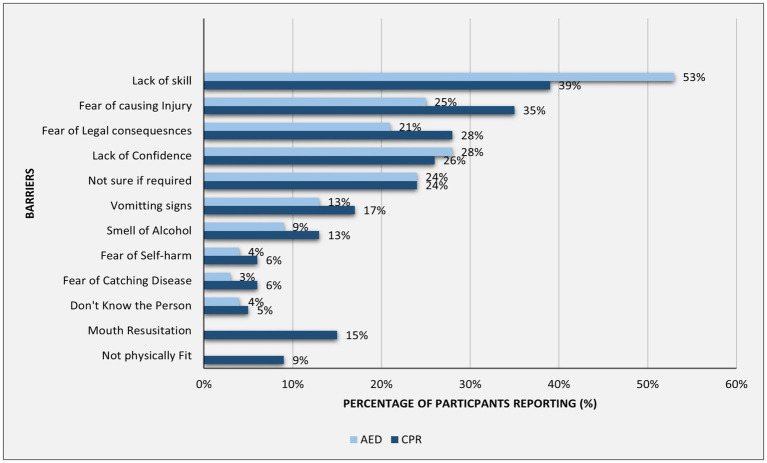
Frequencies of reported barriers to bystander CPR and AED use.

Lack of skill and confidence were more commonly reported for AED (53 and 28%) than for CPR (39 and 26%), while fear of causing injury (35%) and legal concerns (28%) were more prominent for CPR than for AED (25 and 21%) (Figure 2).

**Figure 3:**
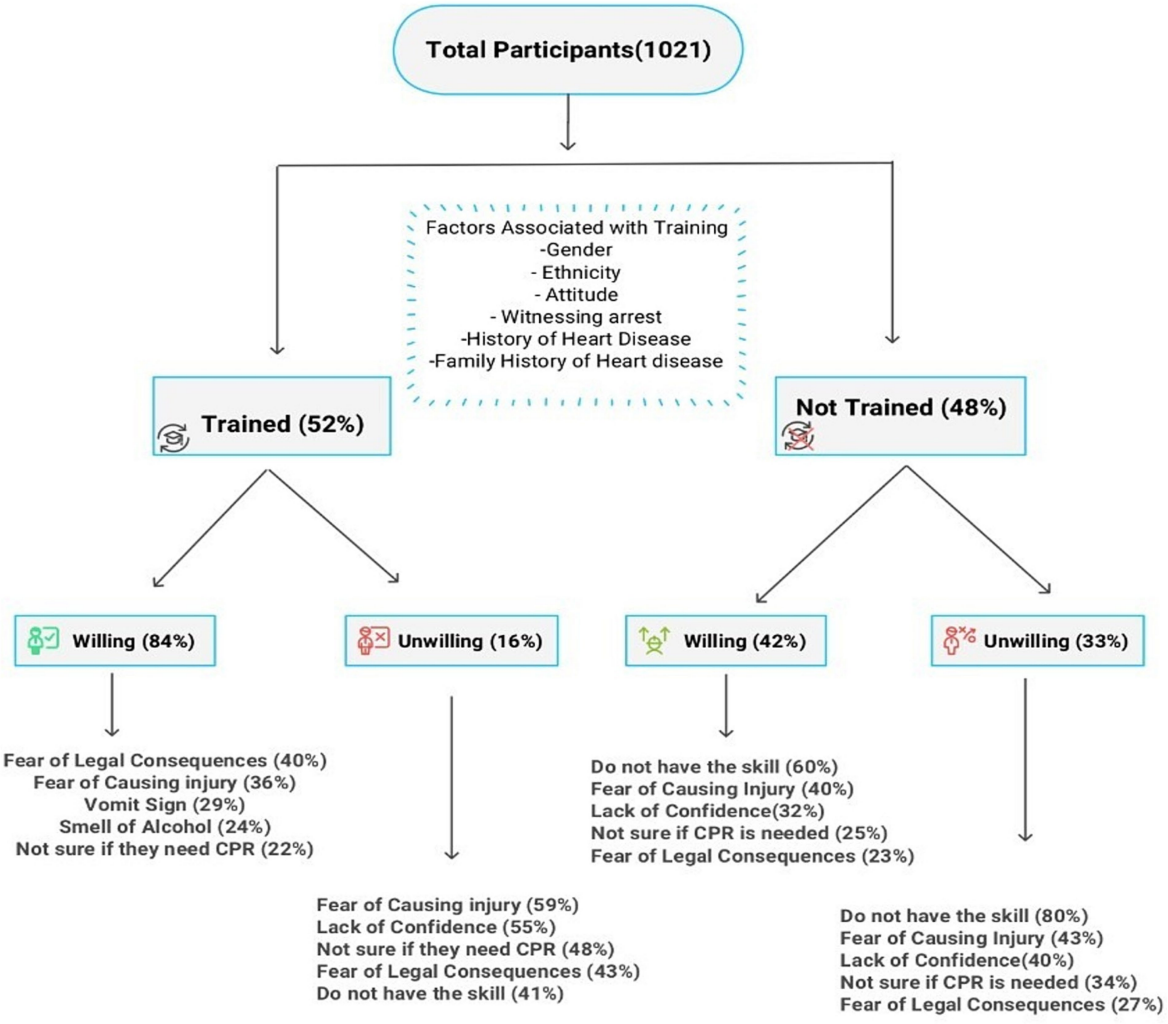
Reported barriers to performing CPR by training status and willingness to act.

Among CPR-trained participants, 16.3% were unwilling to perform CPR, primarily due to fear of harm (59%) and lack of confidence (55%). Among AED-trained respondents, 11.8% were reluctant to intervene due to unfamiliarity and odor of alcohol (both 100%), followed by low confidence (52%) (Figure 3).

**Figure 4:**
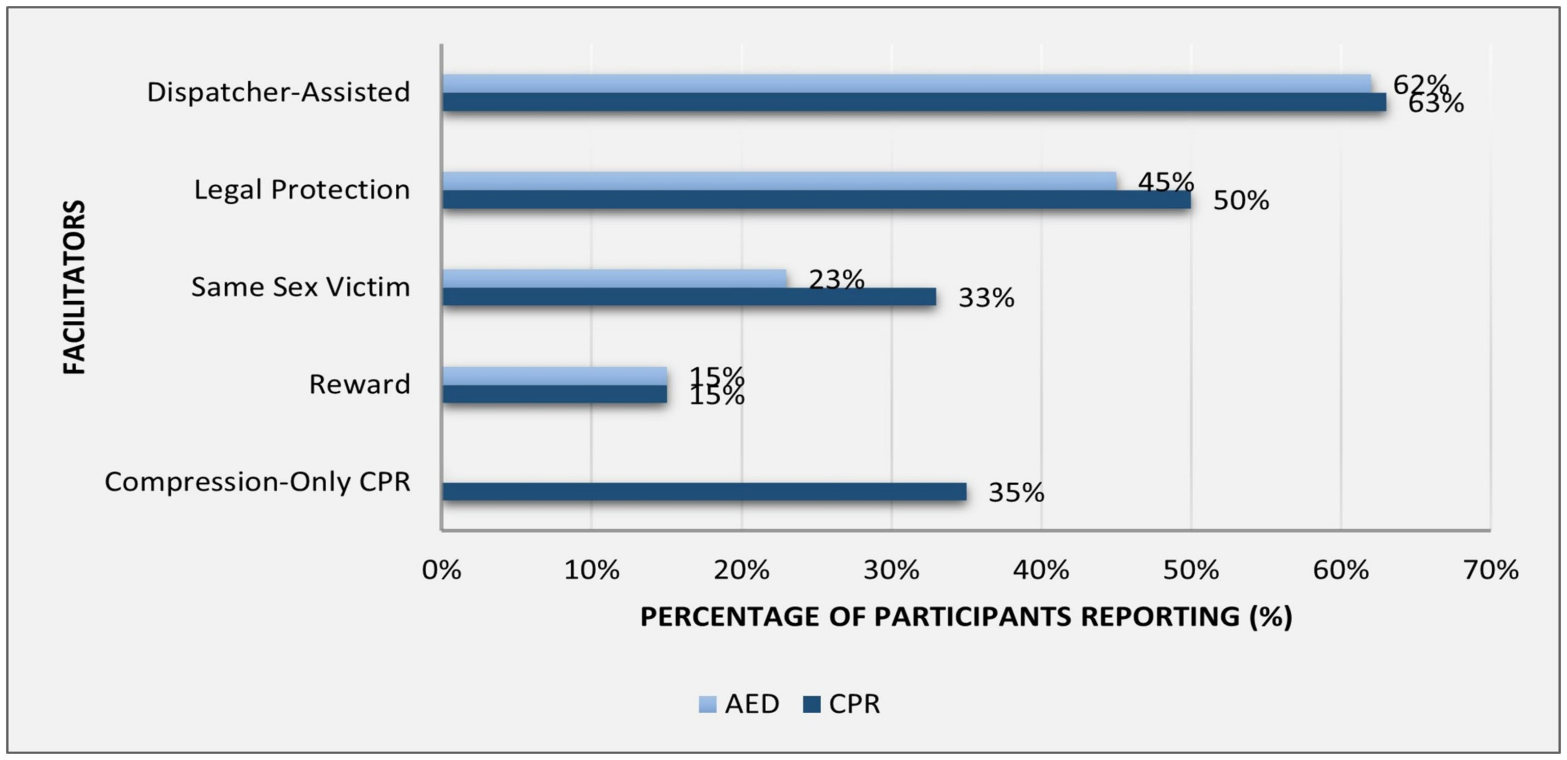
Frequencies of reported facilitators of bystander CPR and use of AED.

Dispatcher-assisted guidance was the top enabler, followed by legal protection (Figure 4).

## 4 Discussion

This study identified the key predictors, barriers, and facilitators to bystander response to an OHCA in the UAE. Lower training rates were observed among females and South Asians, while AED training was also lower among residents of the Northern Emirates and those with lower education. Training was associated with higher confidence and more positive attitudes. Prior training emerged as the strongest predictor of willingness, with repeated training further increasing willingness. Additional predictors included being a healthcare provider, confidence in recognizing cardiac arrest, and belief in the effectiveness of bystander intervention (positive attitude). Bivariate analysis highlighted that more recent and repeated training was linked to higher confidence and greater willingness, demonstrating clear dose–response patterns. Attitudinal and experiential factors, including positive attitudes, the ability to recognize cardiac arrest, and witnessing a cardiac arrest, were additionally associated with higher willingness. Likelihood of performing CPR varied by the sex of the individual in cardiac arrest, with participants more likely to respond to same-sex individuals. Key barriers and facilitators included lack of skill and confidence, fear of causing harm, and legal concerns, and among trained participants who were unwilling to intervene, about half reported low confidence. These findings align with the Theory of Planned Behavior (17), highlighting strong links between willingness and confidence (control beliefs), perceived CPR effectiveness (behavioral beliefs), and prior training (which reinforces both). Social norms such as expectations related to sex and cultural attitudes toward helping strangers reflect the role of normative beliefs. Addressing all three belief domains is essential when designing interventions to promote bystander CPR and AED use.

In our study, 60.6% of participants were willing to perform CPR, and 46.8% were willing to use an AED. Studies have reported varying CPR willingness rates, with higher rates in studies from Taiwan (86.7%) and South Korea (67.5%), compared to lower rates in the UK (57.6%) and Austria (33%) (21–24). Similarly, AED willingness ranges from 43% in a South Korean study to 50% in an Austrian study (24, 25). In our study population, 52% had received CPR training, and 34% had AED training. The CPR training rate in our study was lower than the rates reported from Europe (59%) and North America (65%) but higher than previous studies from the Middle East (19%) (26). AED training rates from our sample matched a recent 2024 survey in Singapore (36%) but were notably higher than the 20% reported in the UK in 2017 (23, 27). However, in a recent study from Qatar, which shares a similar context to UAE, actual bystander CPR was provided in only 34% of out-of-hospital cardiac arrests, underscoring challenges in translating willingness into real-world action (28).

Our findings on training disparities align with previous studies, which have consistently reported lower CPR and AED training rates among certain demographic groups, including South Asians (29), individuals with lower education levels (30), and older adults (30). Females had lower CPR training rates, and although bivariate analysis showed reduced willingness to perform CPR, this was not significant in the multivariable model. Notably, lower willingness among females has been reported in the literature, supporting the relevance of gender as a factor in bystander response (21, 24). Regional variations in AED training rates, identified in our study, warrant further exploration to understand underlying factors. Previous training is a key predictor of willingness to perform CPR, showing a dose-dependent relationship between the number of training sessions and the odds of intervening, including AED use. This aligns with existing literature, which indicates that repeated training enhances willingness (22). Bivariate analysis further demonstrated that willingness decreases as the time since the last training increases, advocating for frequent refresher CPR training sessions. Prior studies have also highlighted skill decay over time, and the need for frequent CPR training sessions has been recommended (31).

Sex influenced CPR willingness based on the affected individual’s characteristics: women were more likely to help women and children, while men preferred assisting male individuals and family members. For AED use, sex differences were not significant when the individual in arrest was male, but men were less likely to use an AED on females. Supporting this, 23% cited same-sex individuals as a facilitator for AED use and 33% for CPR. These findings align with prior research showing women are less likely to receive CPR due to fears of inappropriate touch or accusations of sexual assault (30, 32).

The most reported barriers to CPR and AED use were lack of skill, confidence, uncertainty, fear of injury, and legal consequences, consistent with other studies (33). The most frequently cited barrier among trained individuals who expressed willingness to perform CPR was the fear of legal consequences. This underscores the critical need for increased public awareness regarding the Good Samaritan Law, which was enacted in the UAE in November 2020 (34). Notably, 50% of respondents identified legal protection as a key motivator for initiating CPR, emphasizing the law’s potential role in encouraging bystander intervention. The perception of legal concerns as a barrier varies across studies, potentially due to differing legislative frameworks and levels of public awareness (21, 33). Furthermore, among participants who were unwilling to perform CPR despite training, 55% cited a lack of confidence in their CPR skills, and 52% lacked confidence in using an AED. This highlights the importance of training programs that enhance both technical skills and psychological readiness.

Dispatcher-assisted CPR (DA-CPR) was identified as a key facilitator in our study, consistent with findings from the UK, where 82% of participants reported confidence if an emergency responder guides them (35). Around 35% of participants identified compression-only CPR (CO-CPR) as a facilitator. In another study, 61% preferred CO-CPR on unknown individuals, which means significant bystander CPR rates may be increased by promoting CO- CPR (33, 36).

Based on our findings, several targeted interventions are needed to improve bystander response to OHCA. Efforts to improve training rates could emphasize female inclusivity and outreach to underrepresented ethnic groups, especially South Asians. Variations across emirates should be further explored using more diverse samples to improve accessibility. Frequent, repeated training integrated into school curricula, linked to driver’s licenses, and supported by CPR self-training kits can increase training rates and bystander response, as seen in Denmark, where such measures doubled bystander CPR (37). Promoting DA-CPR and CO-CPR can further boost intervention. Cultural and religious concerns, particularly CPR on opposite-sex, culd be addressed through engagement with the community and religious leaders and legal concerns could be addressed by raising awareness about Good Samaritan laws. According to the willingness-centered bystander model, an individual’s decision to help is shaped by personal beliefs, social norms, and confidence in predicting the outcome of their actions (38), as demonstrated in our study, where a positive attitude was strongly associated with the willingness to perform CPR. These findings suggest that promoting positive beliefs about the potential life-saving impact of bystander CPR and AED use may support greater willingness to intervene.

### 4.1 Limitations

The limitations of this study should be considered when interpreting the results. First, the cross-sectional design precludes causal inference. Second, convenience sampling may introduce selection bias, as more accessible or more willing to participate might not represent the broader population. Additionally, recruitment sources such as university mailing lists compared to public locations may have resulted in differing probabilities of CPR training exposure among participants. Third, our sample demonstrated a skewed regional and demographic distribution, with an overrepresentation of younger, educated Emiratis primarily from Al Ain and Abu Dhabi, which may limit the generalizability of our findings to the wider UAE population. Notably, South Asians, who make up approximately 50% of the UAE population, were underrepresented (9%) in our sample (39). Fourth, for participants trained two or three times, estimates showed wide confidence intervals, reflecting limited precision for these categories. Fifth, recall imprecision may influence self-reported training frequency and recency of training and measures such as confidence in performing CPR and ability to recognize cardiac arrest were also self-reported rather than objectively assessed. Finally, while behavioral intentions predict actions, their concordance with actual responses to out-of-hospital cardiac arrest remains uncertain.

## 5 Conclusion

Despite the Good Samaritan Law and existing CPR training programs, significant barriers to bystander intervention remain in the UAE. Our findings suggest that inclusive and culturally sensitive training, along with measures addressing behavioral barriers related to legal concerns and social norms, may help support bystander response in out-of-hospital cardiac arrest (OHCA) situations. Supportive approaches, such as dispatcher-assisted CPR and compression-only CPR, were also identified as potential facilitators. Representative national estimates of training and actual bystander CPR and AED use are needed. Furthermore qualitative research is needed to gain a deeper understanding of the social, cultural, and legal barriers affecting both trained and untrained bystanders.

## Data Availability

The raw data supporting the conclusions of this article will be made available by the authors, without undue reservation.
