# Supplementary material for: Predictors, barriers and facilitators of bystander interventions in out of hospital cardiac arrest: a cross-sectional study from the UAE
Source: Front Public Health. 2026 Feb 23;14:1738145. doi: 10.3389/fpubh.2026.1738145 (PMC12968197; doi:10.3389/fpubh.2026.1738145)
Supplement: Supplementary file 1 [file Data_Sheet_1.docx]

**Appendix A**

Table of Contents

Table A1. Association Between CPR Training Status and Participants’ Characteristics ……….2

Table A2: Association Between AED Training Status and Participants’ Characteristics ………4

Table A3: Association Between Willingness to Perform CPR and Participant Characteristics…6

Table A4: Association Between Willingness to Use an AED and Participant Characteristics….8

Table A5: Univariate Analysis for willingness to perform CPR and Use AED ….10

# Table A1. Association Between CPR Training Status and Participants’ Characteristics

| Characteristic | Categories | Not Trained (N =222)^1^ | Trained (N =459) | p- value^2^ |
| --- | --- | --- | --- | --- |
| Age |  |  |  | 0.90 |
|  | 18-35 | 311 (48%) | 342 (52%) |  |
|  | 36-49 | 84 (48%) | 91 (52%) |  |
|  | 50 and above | 27 (51%) | 26 (49%) |  |
|  |  |  |  |  |
| Gender |  |  |  | **0.003** |
|  | Female | 265 (52%) | 243 (48%) |  |
|  | Male | 157 (42%) | 216 (58%) |  |
|  |  |  |  |  |
| Location |  |  |  | 0.11 |
|  | Abu Dhabi | 129 (43%) | 168 (57%) |  |
|  | Al Ain | 218 (50%) | 221 (50%) |  |
|  | Dubai | 27 (44%) | 34 (56%) |  |
|  | Other (Ajman, Sharjah, Ras al Khaimah, Fujairah, Umm Al-Quwain) | 48 (57%) | 36 (43%) |  |
|  |  |  |  |  |
| Ethnicity |  |  |  | **<0.001** |
|  | Emiratis | 267 (44%) | 345 (56%) |  |
|  | Other Arabs | 67 (50%) | 68 (50%) |  |
|  | South Asians (Pakistanis, Bangladeshis, Indians) | 69 (78%) | 20 (22%) |  |
|  | Southeast Asians (Filipinos, Indonesians) | 3 (30%) | 7 (70%) |  |
|  | African | 10 (63%) | 6 (38%) |  |
|  | Western | 6 (32%) | 13 (68%) |  |
|  |  |  |  |  |
| Education level |  |  |  | 0.20 |
|  | Primary/Secondary School | 93 (52%) | 86 (48%) |  |
|  | College/University (bachelor’s degree or equivalent) | 246 (48%) | 266 (52%) |  |
|  | Postgraduate | 79 (43%) | 104 (57%) |  |
|  |  |  |  |  |
| Personal History of Heart Disease |  |  |  | **<0.001** |
|  | No | 405 (50%) | 410 (50%) |  |
|  | Yes | 17 (26%) | 49 (74%) |  |
| Family History of Heart Disease |  |  |  | **<0.001** |
|  | No | 289 (52%) | 264 (48%) |  |
|  | Yes | 133 (41%) | 195 (59%) |  |
|  |  |  |  |  |
| Residing with family member aged >65 |  |  |  | 0.30 |
|  | No | 237 (50%) | 241 (50%) |  |
|  | Yes | 185 (46%) | 218 (54%) |  |
|  |  |  |  |  |
| Believe that bystander CPR can increase survival (positive attitude) |  |  |  | **<0.001** |
|  | No | 39 (81%) | 9 (19%) |  |
|  | Yes | 383 (46%) | 450 (54%) |  |
|  |  |  |  |  |
| Witnessed Arrest |  |  |  | **<0.001** |
|  | Yes | 334 (53%) | 302 (47%) |  |
|  | No | 36 (22%) | 129 (78%) |  |
|  |  |  |  |  |
| Confident in ability to perform CPR |  |  |  | **<0.001** |
|  | Not Confident | 234 (85%) | 40 (15%) |  |
|  | Somewhat Confident | 119 (48%) | 127 (52%) |  |
|  | Confident | 38 (20%) | 150 (80%) |  |
|  | Very Confident | 31 (18%) | 142 (82%) |  |
| ^1^ n (%)  ^2^ Pearson’s Chi-squared test/ Fisher’s Exact Test |  |  |  |  |
|  |  |  |  |  |

# Table A2: Association Between AED Training Status and Participants’ Characteristics

| Characteristic | Categories | Not Trained (N =222)^1^ | Trained (N =459) | P- value^2^ |
| --- | --- | --- | --- | --- |
| Age |  |  |  | 0.055 |
|  | 18-35 | 404 (66%) | 204 (34%) |  |
|  | 36-49 | 96 (60%) | 63 (40%) |  |
|  | 50 and above | 40 (78%) | 11 (22%) |  |
|  |  |  |  |  |
| Gender |  |  |  | 0.30 |
|  | Female | 311 (67%) | 150 (33%) |  |
|  | Male | 229 (64%) | 128 (36%) |  |
|  |  |  |  |  |
| Location |  |  |  |  |
|  | Abu Dhabi | 170 (60%) | 115 (40%) | **0.006** |
|  | Al Ain | 270 (68%) | 126 (32%) |  |
|  | Dubai | 38 (64%) | 21 (36%) |  |
|  | Other (Ajman, Sharjah, Ras al Khaimah, Fujairah, Umm Al-Quwain) | 62 (79%) | 16 (21%) |  |
|  |  |  |  |  |
| Ethnicity |  |  |  | **<0.001** |
|  | Emiratis | 355 (63%) | 208 (37%) |  |
|  | Other Arabs | 83 (66%) | 42 (34%) |  |
|  | South Asians (Pakistanis, Bangladeshis, Indians) | 77 (89%) | 10 (11%) |  |
|  | Southeast Asians (Filipinos, Indonesians) | 5 (50%) | 5 (50%) |  |
|  | African | 7 (50%) | 7 (50%) |  |
|  | Western |  |  |  |
|  |  |  |  |  |
| Education level |  |  |  |  |
|  | Primary/Secondary School | 121 (72%) | 47 (28%) | **0.003** |
|  | College/University (bachelor’s degree or equivalent) | 319 (68%) | 153 (32%) |  |
|  | Postgraduate | 96 (55%) | 77 (45%) |  |
|  |  |  |  |  |
| Personal History of Heart Disease |  |  |  | **<0.001** |
|  | No | 520 (69%) | 234 (31%) |  |
|  | yes | 20 (31%) | 44 (69%) |  |
| Family History of Heart Disease |  |  |  | **<0.001** |
|  | No | 358 (70%) | 151 (30%) |  |
|  | Yes | 182 (59%) | 127 (41%) |  |
|  |  |  |  |  |
| Residing with family member aged >65 |  |  |  | 0.40 |
|  | No | 299 (67%) | 146 (33%) |  |
|  | Yes | 241 (65%) | 132 (35%) |  |
|  |  |  |  |  |
| Witnessed Arrest |  |  |  | **<0.001** |
|  | No | 481 (76%) | 155 (24%) |  |
|  | Yes | 48 (29%) | 117 (71%) |  |
|  |  |  |  |  |
| Confident in ability to perform CPR |  |  |  | **<0.001** |
|  | Not Confident | 363 (96%) | 15 (4.0%) |  |
|  | Somewhat Confident | 97 (73%) | 35 (27%) |  |
|  | Confident | 55 (38%) | 90 (62%) |  |
|  | Very Confident | 25 (15%) | 138 (85%) |  |
| ^1^ n (%)  ^2^ Pearson’s Chi-squared test/ Fisher’s Exact Test |  |  |  |  |
|  |  |  |  |  |

# Table A3. Associations Between Willingness to Perform CPR and Participant Characteristics

| **Characteristics** | **Categories** | **Not Willing (n=401)^1^** | **Willing (n=619)^2^** | **p-value** |
| --- | --- | --- | --- | --- |
| Age | 18-35 | 311 (40%) | 460 (60%) | 0.30 |
|  | 36-49 | 73 (38%) | 119 (62%) |  |
|  | 50 and above | 17 (30%) | 40 (70%) |  |
|  |  |  |  |  |
| Gender | Female | 267 (44%) | 344 (56%) | **<0.001** |
|  | Male | 134 (33%) | 275 (67%) |  |
|  |  |  |  |  |
| Marital status | Married | 108 (33%) | 217 (67%) | **0.013** |
|  | Unmarried | 280 (43%) | 376 (57%) |  |
|  | Divorced/ Widowed | 13 (33%) | 26 (67%) |  |
|  |  |  |  |  |
| Education level | Primary/Secondary School | 91 (43%) | 121 (57%) | **0.044** |
|  | College/University (bachelor’s degree or equivalent) | 243 (41%) | 356 (59%) |  |
|  | Postgraduate | 64 (32%) | 137 (68%) |  |
|  |  |  |  |  |
| Income level (AED) | < 30,000 AED | 137 (34%) | 270 (66%) | 0.70 |
|  | >30,000 AED | 45 (32%) | 97 (68%) |  |
|  |  |  |  |  |
| Location | Abu Dhabi | 106 (33%) | 220 (67%) | **<0.001** |
|  | Al ain | 197 (39%) | 304 (61%) |  |
|  | Dubai | 32 (43%) | 43 (57%) |  |
|  | Northern Emirates (Sharjah, Fujairah, Ras al Khaima, Ajman, Umm Al-Quwain) | 66 (56%) | 52 (44%) |  |
|  |  |  |  |  |
| Ethnicity | Emiratis | 295 (41%) | 432 (59%) | 0.60 |
|  | Other Arabs | 54 (36%) | 98 (64%) |  |
|  | South Asians | 33 (36%) | 59 (64%) |  |
|  | Others | 19 (39%) | 30 (61%) |  |
|  |  |  |  |  |
| Healthcare Provider | No | 356 (45%) | 437 (55%) | **<0.001** |
|  | yes | 45 (20%) | 182 (80%) |  |
|  |  |  |  |  |
| Trained | No | 187 (44%) | 235 (56%) | **<0.001** |
|  | yes | 75 (16%) | 384 (84%) |  |
|  |  |  |  |  |
| Training Frequency | 1 time | 49 (31%) | 110 (69%) | **<0.001** |
|  | 2 times | 14 (13%) | 90 (87%) |  |
|  | 3 times or more | 10 (6.3%) | 149 (94%) |  |
|  |  |  |  | **<0.001** |
| Times since last trained | More than 5 years ago | 22 (32%) | 47 (68%) |  |
|  | More than 1 year ago but within 5 years | 27 (18%) | 124 (82%) |  |
|  | Less than one year ago | 23 (11%) | 181 (89%) |  |
|  |  |  |  |  |
| Positive attitude towards CPR | No | 29 (54%) | 25 (46%) | **0.002** |
|  | Yes | 294 (33%) | 594 (67%) |  |
|  |  |  |  |  |
| History of heart Disease | No | 355 (39%) | 567 (61%) | **0.033** |
|  | Yes | 18 (26%) | 52 (74%) |  |
|  |  |  |  |  |
| Family history of heart disease | No | 247 (39%) | 380 (61%) | 0.10 |
|  | Yes | 126 (35%) | 239 (65%) |  |
|  |  |  |  |  |
| Residing with family member aged >65 | No | 205 (37%) | 345 (63%) | 0.80 |
|  | Yes | 168 (38%) | 274 (62%) |  |
|  |  |  |  | **<0.001** |
| Ability to recognize cardiac arrest | No | 244 (46%) | 285 (54%) |  |
|  | Yes | 102 (23%) | 334 (77%) |  |
|  |  |  |  |  |
| Confidence in performing CPR | Not Confident | 162 (59%) | 112 (41%) | **<0.001** |
|  | Somewhat confident | 65 (26%) | 181 (74%) |  |
|  | Confident | 23 (12%) | 165 (88%) |  |
|  | Very Confident | 12 (6.9%) | 161 (93%) |  |
|  |  |  |  |  |
| Witnessed cardiac arrest | No | 216 (34%) | 420 (66%) | **<0.001** |
|  | Yes | 22 (13%) | 143 (87%) |  |
|  |  |  |  |  |
| ^1^n (%) ^2^Pearson’s Chi-squared test |  |  |  |  |

# Table A4. Associations Between Willingness to Use an AED and Participant Characteristics

| **Characteristics** | **Categories** | **Not Willing (n=)^1^** | **Willing (n=)^2^** | **p-value** |
| --- | --- | --- | --- | --- |
| Age | 18-35 | 417 (54%) | 354 (46%) | 0.60 |
|  | 36-49 | 97 (51%) | 95 (49%) |  |
|  | 50 and above | 28 (49%) | 29 (51%) |  |
|  |  |  |  |  |
| Gender | Female | 339 (55%) | 272 (45%) | 0.067 |
|  | Male | 203 (50%) | 206 (50%) |  |
|  |  |  |  |  |
| Marital status | Married | 157 (48%) | 168 (52%) | 0.093 |
|  | Unmarried | 365 (56%) | 291 (44%) |  |
|  | Divorced/ Widowed | 20 (51%) | 19 (49%) |  |
|  |  |  |  |  |
| Education level | Primary/Secondary School | 132 (62%) | 80 (38%) | **<0.001** |
|  | College/University (bachelor’s degree or equivalent) | 317 (53%) | 282 (47%) |  |
|  | Postgraduate | 88 (44%) | 113 (56%) |  |
|  |  |  |  |  |
| Income level (AED) | < 30,000 AED | 206 (51%) | 201 (49%) | 0.086 |
|  | >30,000 AED | 60 (42%) | 82 (58%) |  |
|  |  |  |  |  |
| Location | Abu Dhabi | 161 (49%) | 165 (51%) | **0.048** |
|  | Al ain | 266 (53%) | 235 (47%) |  |
|  | Dubai | 39 (52%) | 36 (48%) |  |
|  | Northern Emirates (Sharjah, Fujairah, Ras al Khaima, Ajman, Umm Al-Quwain) | 76 (64%) | 42 (36%) |  |
|  |  |  |  |  |
| Ethnicity | Emiratis | 377 (52%) | 350 (48%) | 0.11 |
|  | Other Arabs | 79 (52%) | 73 (48%) |  |
|  | South Asians | 60 (65%) | 32 (35%) |  |
|  | Others | 26 (53%) | 23 (47%) |  |
|  |  |  |  |  |
| Healthcare Provider | No | 475 (60%) | 318 (40%) | <0.001 |
|  | yes | 67 (30%) | 160 (70%) |  |
|  |  |  |  | **<0.001** |
| Trained | No | 307 (57%) | 233 (43%) |  |
|  | yes | 33 (12%) | 245 (88%) |  |
|  |  |  |  |  |
| Training Frequency | 1 time | 15 (19%) | 62 (81%) | **0.045** |
|  | 2 times | 5 (7.7%) | 60 (92%) |  |
|  | 3 times or more | 8 (8.7%) | 84 (91%) |  |
|  |  |  |  |  |
| Times since last trained | More than 5 years ago | 13 (8.6%) | 139 (91%) | **0.005** |
|  | More than 1 year ago but within 5 years | 5 (42%) | 7 (58%) |  |
|  | Less than one year ago | 12 (16%) | 64 (84%) |  |
|  |  |  |  |  |
| Positive attitude towards CPR | No | 40 (74%) | 14 (26%) | **<0.001** |
|  | Yes | 424 (48%) | 464 (52%) |  |
|  |  |  |  |  |
| History of heart Disease | No | 493 (53%) | 429 (47%) | **<0.001** |
|  | Yes | 21 (30%) | 49 (70%) |  |
|  |  |  |  |  |
| Family history of heart disease | No | 348 (56%) | 279 (44%) | **0.002** |
|  | Yes | 166 (45%) | 199 (55%) |  |
|  |  |  |  |  |
| Residing with family member aged >65 | No | 301 (55%) | 249 (45%) | **0.041** |
|  | Yes | 213 (48%) | 229 (52%) |  |
|  |  |  |  | **<0.001** |
| Ability to recognize cardiac arrest | No | 288 (61%) | 181 (39%) |  |
|  | Yes | 199 (40%) | 297 (60%) |  |
|  |  |  |  |  |
| Confidence in performing CPR | Not Confident | 269 (71%) | 109 (29%) | **<0.001** |
|  | Somewhat confident | 40 (30%) | 92 (70%) |  |
|  | Confident | 19 (12%) | 144 (88%) |  |
|  | Very Confident | 12 (8.3%) | 133 (92%) |  |
|  |  |  |  |  |
| Witnessed cardiac arrest | No |  |  | **<0.001** |
|  | Yes |  |  |  |
| ^1^n (%) ^2^Pearson’s Chi-squared test | | | | |

# Table A5. Univariate Analysis from logistic regression for willingness to perform CPR and Use AED

|  | | **CPR Willingness** | | **AED Willingness** | |
| --- | --- | --- | --- | --- | --- |
| **Characteristics** | **Categories** | **aOR (95% CI)** | **p-value** | **aOR (95% CI)** | **p-value** |
| Age | 18-35 | ref |  | ref |  |
|  | 36-49 | 1.10 (0.80, 1.53) | 0.6 | 1.15 (0.84, 1.58) | 0.4 |
|  | 50 and above | 1.59 (0.90, 2.93) | 0.12 | 1.22 (0.71, 2.10) | 0.5 |
|  |  |  |  |  |  |
| Gender | Female | ref |  | ref |  |
|  | Male | 1.59 (1.23, 2.07) | **<0.001** | 1.26 (0.98, 1.63) | 0.067 |
|  |  |  |  |  |  |
| Marital status | Married | ref |  | ref |  |
|  | Unmarried | 0.67 (0.51, 0.88) | **0.004** | 0.75 (0.57, 0.97) | **0.030** |
|  | Divorced/ Widowed | 1.00 0.50, 2.07 | >0.9 | 0.89 (0.45, 1.73) | 0.7 |
|  |  |  |  |  |  |
| Education level | Primary/Secondary School | ref |  | ref |  |
|  | College/University (bachelor’s degree or equivalent) | 1.10 (0.80, 1.51) | 0.5 | 1.47 (1.07, 2.03) | **0.019** |
|  | Postgraduate | 1.61 (1.08, 2.41) | **0.020** | 2.12 (1.43, 3.15) | **<0.001** |
|  |  |  |  |  |  |
| Income level (AED) | < 30,000 AED | ref |  | ref |  |
|  | >30,000 AED | 1.09 (0.73, 1.66) | 0.7 | 1.40 (0.95, 2.07) | 0.087 |
|  |  |  |  |  |  |
| Location | Abu Dhabi | ref |  | ref |  |
|  | Al Ain | 0.74 0.55, 1.00 | 0.047 | 0.86 (0.65, 1.14) | 0.3 |
|  | Dubai | 0.65 0.39, 1.09 | 0.10 | 0.90 (0.54, 1.49) | 0.7 |
|  | Northern Emirates (Sharjah, Fujairah, Ras al Khaima, Ajman, Umm Al-Quwain) | 0.38 0.25, 0.58 | **<0.001** | 0.54 (0.35, 0.83) | 0.005 |
|  |  |  |  |  |  |
| Ethnicity | Emiratis | ref |  | ref |  |
|  | Other Arabs | 1.24 (0.86, 1.79) | 0.2 | 1.00 (0.70, 1.41) | >0.9 |
|  | South Asians | 1.22 (0.78, 1.93) | 0.4 | 0.57 (0.36, 0.90) | **0.016** |
|  | Others | 1.08 0.60, 1.98 | 0.8 |  |  |
|  |  |  |  |  |  |
| Healthcare Provider | No | ref |  | ref |  |
|  | yes | 3.29 (2.33, 4.75) | **<0.001** | 3.57 (2.60, 4.93) | **<0.001** |
| Employment | Employed | ref |  |  |  |
|  | Unemployed | 0.65 (0.36, 1.16) | 0.060 | 0.92 (0.51, 1.65) | 0.8 |
|  | Students | 0.72 (0.52, 1.01) | 0.14 | 0.87 (0.63, 1.22) | 0.4 |
| Training | Not Trained | ref |  | ref |  |
|  | 1 time | 1.79 (1.22, 2.6) | **0.003** | 5.45 (3.10, 10.2) | **<0.001** |
|  | 2 times | 5.12 (2.91, 9.6) | **<0.001** | 15.8 (6.88, 45.8) | **<0.001** |
|  | 3 times or more | 11.9 (6.38, 24.6) | **<0.001** | 13.8 (6.97, 31.5) | **<0.001** |
|  |  |  |  |  |  |
| Times since last trained | More than 5 years ago | ref |  | ref |  |
|  | More than 1 year ago but within 5 years | 2.15 (1.11, 4.14) | **0.022** | 7.64 (2.02, 27.6) | **0.002** |
|  | Less than one year ago | 3.68 (1.89, 7.21) | <0.001 | - | - |
|  |  |  |  |  |  |
| Positive attitude towards CPR | No | ref |  | ref |  |
|  | Yes | 2.34 (1.35, 4.10) | **0.003** | 3.13(1.72, 6.03) | **<0.001** |
|  |  |  |  |  |  |
| History of heart Disease | No | ref |  | ref |  |
|  | Yes | 1.81 (1.06, 3.22) | **0.035** | 2.68 (1.60, 4.63) | **<0.001** |
|  |  |  |  |  |  |
| Family history of heart disease | No | ref |  | ref |  |
|  | Yes | 1.23 (0.94, 1.62) | 0.10 | 1.50 (1.15, 1.94) | **0.002** |
|  |  |  |  |  |  |
| Residing with family member aged >65 | No | ref |  | ref |  |
|  | Yes | 0.97 (0.75, 1.26) | 0.8 | 1.30 (1.01, 1.67) | **0.041** |
|  |  |  |  |  |  |
| Ability to recognize cardiac arrest | No | ref |  | ref |  |
|  | Yes | 2.80 (2.12, 3.72) | **<0.001** | 2.79 (2.15, 3.63) | **<0.001** |
|  |  |  |  |  |  |
| Confidence in performing CPR | Not Confident | ref |  | ref |  |
|  | Somewhat confident | 4.03 (2.79, 5.87) | **<0.001** | 5.68 (3.71, 8.82) | **<0.001** |
|  | Confident | 10.4 (6.41, 17.4) | **<0.001** | 27.4 (15.1, 54.0) | **<0.001** |
|  | Very Confident | 19.4 (10.7, 38.4) | **<0.001** | 18.7 11.3, 32.6 | **<0.001** |
|  |  |  |  |  |  |
| Witnessed cardiac arrest | No | ref |  | ref |  |
|  | Yes | 3.34 (2.11, 5.52) | **<0.001** | 3.03 (2.06, 4.56) | **<0.001** |
