# Supplementary material for: Predictors, barriers and facilitators of bystander interventions in out of hospital cardiac arrest: a cross-sectional study from the UAE
Source: Front Public Health. 2026 Feb 23;14:1738145. doi: 10.3389/fpubh.2026.1738145 (PMC12968197; doi:10.3389/fpubh.2026.1738145)
Supplement: Supplementary file 2 [file Data_Sheet_2.docx]

Contents

[**Checklist for Reporting Results of Internet E-Surveys (CHERRIES)** 2](#_Toc200555560)

[Survey Questionnaire 5](#_Toc200555561)

[English Version 5](#_Toc200555562)

[Arabic Version 13](#_Toc200555563)

[Urdu Version 19](#_Toc200555564)

# **Checklist for Reporting Results of Internet E-Surveys (CHERRIES)**

| **Item Category** | **Checklist Item** | **Explanation** |
| --- | --- | --- |
| Design | Describe survey design | A cross-sectional design was used. The target population included adults (≥18 years) who were nationals or residents of the UAE. Convenience and snowball sampling were employed. |
| IRB approval and informed consent process | IRB approval | The study was approved by the UAE University Ethics Board (ERSC_2024_4460). |
|  | Informed consent | Participants provided informed consent. The purpose of the study, data handling, and investigator details were described. |
|  | Data protection | No personal identifiable data were collected. Data were stored securely and accessed only by the research team. |
| Development and pre-testing | Development and testing | The survey was developed based on literature and validated by experts. Translated into Arabic and Urdu and backtranslated. |
| Recruitment process and description of the sample having access to the questionnaire | Open survey versus closed survey | Open survey – participation was voluntary. |
|  | Contact mode | Survey was distributed both in person and online (email/social media). |
|  | Advertising the survey | Advertised via university mailing lists |
| Survey administration | Web/E-mail | Administered via in-person tablet-based entry and online using email/social media links. |
|  | Context | University and healthcare-related platforms and public venues. |
|  | Mandatory/voluntary | Participation was voluntary. |
|  | Incentives | No incentives were offered. |
|  | Time | Data collected during July 2024 to November 2024 |
|  | Randomization of items or questionnaires | Not applied. |
|  | Adaptive questioning | Not applied. |
|  | Number of Items | 35 items. |
|  | Number of screens (pages) | Displayed 6 pages. |
|  | Completeness check | Mandatory questions marked with asterisks and required for submission. This ensured that respondents could not proceed without providing an answer to those questions, thus minimizing missing data at the point of entry. |
|  | Review step | Respondents could go back and review and change answers before submitting |
| Response rates | Unique site visitor | SurveyMonkey does track unique survey responses internally and can prevent multiple submissions from the same browser using cookies (if enabled), but it does not provide detailed site visitor analytics |
|  | View rate | Not applicable. |
|  | Participation rate | Not applicable. |
|  | Completion rate | Completion rate—defined as the number of participants who submitted the final page of the questionnaire divided by those who agreed to participate or accessed the first survey page—could not be accurately measured in our case. This is because we did not use a separate “informed consent” page; consent was implied by proceeding with the survey. |
| Preventing multiple entries from the same individual | Cookies used | Not used. |
|  | IP check | To avoid duplicate entries, a restriction was placed within survey monkey to block multiple submissions from the same IP address for online responses. For in-person data collection using tablets, this restriction was removed to allow multiple respondents to use the same device |
|  | Log file analysis | Not performed. |
|  | Registration | Not applicable. |
| Analysis | Handling of incomplete questionnaires | The overall missingness in our data was fairly minimal at about 7%. Moreover, we do not have missing data in the outcome variables. |
|  | Questionnaires submitted with an atypical timestamp | Not excluded. No automated time cut off applied. |
|  | Statistical correction | For descriptive and univariate analyses, we used pairwise deletion, ensuring the maximum utilization of the available data. For multivariable logistic regression models, listwise deletion was applied by default using R’s glm() function to address missing data. |

# **Survey Questionnaire**

## English Version

Demographics

1. What is your age?

2. What is your gender?

- Male
- Female

3. What is your marital status?

- Married
- Unmarried
- divorced/widowed

4. Where are you located?

- Abu Dhabi
- Al-Ain
- Dubai
- Sharjah
- Ajman
- Umm Al-Quwain
- Ras Al Khaimah
- Fujairah

5. What is your ethnicity?

- Emirati
- Other Arab
- South Asian (e.g., Indian, Pakistani, Bangladeshi) South East Asian (e.g., Filipino, Indonesian)
- Western
- African
- Other (please specify)

6. What is your education level?

- Primary/Secondary School
- College/University (Bachelor's degree or equivalent)
- Postgraduate School (Master's degree or equivalent)
- Advanced Postgraduate School (Ph.D., MD, JD, etc.) Other (please specify)

7. Are you a Healthcare provider (doctor/nurse/dentist/para-medic)

- Yes
- No

8. What is your current occupation? Please select one of the following categories:

- Healthcare worker
- Teacher/Educator
- Science and research
- IT/software engineer
- Administrative staff
- Legal and Law Enforcement
- Real Estate and Property Management Human Resources
- Finance and accounting
- Non-Profit/ Social Service
- Construction/Manual labor
- Retail/Sales or Marketing
- Hospitality/Service industry
- Student
- Unemployed
- Other (please specify)

9. What type of workplace do you work in? Please select one of the following categories:

- Healthcare facility (e.g., hospital, clinic)
- School/Educational institution
- Office/Corporate setting Laboratory
- Construction site
- Manufacturing/Industrial Site
- Hospitality Venue (e.g., hotel, restaurant)
- Transportation Hub (e.g., airport, train station) Retail store
- Restaurant/Café
- Non-Profit Organization Remote work
- Law enforcement/ Security forces Home
- Unemployed Other (please specify)

10. What is your monthly Income?

- Unemployed
- <4999 AED
- 5000-9999 AED
- 10,000- 14,999 AED
- 15,000-19,999 AED
- 20,000- 24,999 AED
- 25,000-29,999 AED
- 30,000-34,999 AED
- 36,000 - 39,999 AED
- 40,000-44,999 AED
- 45,000-49,999 AED
- >50,000

**Personal and Family History**

11. Are YOU suffering from heart disease? (Angina, myocardial infarction, stroke, other cardiac disorders, cardiac surgery/procedure done)

- Yes
- No

12. Is there any family member suffering from heart disease? (Angina, myocardial infarction stroke, other cardiac disorders, cardiac surgery/procedure done)

- Yes
- No

13. Do you have a family elder older than 65 years old, living with you?

- Yes
- No

Cardiac Arrest

14. Do you feel confident in your ability to recognize if someone is suffering cardiac arrest?

- Yes
- No

15. Do you feel confident in your ability to recognize if somebody is suffering heart attack?

- Yes
- No

16. If you suspect someone is experiencing cardiac arrest, what is the correct emergency number to call for medical help in UAE?

- 911
- 999
- 112
- 998

**Attitudes**

Cardiopulmonary resuscitation (CPR) is an emergency procedure that is performed on a person suffering a cardiac arrest; it involves chest compressions to maintain circulation until an ambulance arrives with OR without rescue breathing (i.e. mouth- to-mouth resuscitation)

17. Do you believe CPR provided by a bystander until Emergency Medical Service (EMS) arrives can increase the chances of survival of the victim?

- Yes
- No

18. Do you believe the general public should be taught the skills to perform CPR?

- Yes
- No

The next few questions are related to CPR (Cardiopulmonary resuscitation). Please select the option that best reflects your response to the statements below.

Cardiopulmonary resuscitation (CPR) is an emergency procedure that is performed on a person suffering a cardiac arrest; it involves chest compressions to maintain circulation until an ambulance arrives with OR without rescue breathing (i.e. mouth- to-mouth resuscitation)

19. Do you feel confident in your ability to perform CPR?

- Very confident
- Confident
- Somewhat Confident
- Not Confident

20. Have you ever been trained/taken sessions for CPR?

- Yes
- No

21. How many times have you been trained/taken CPR sessions?

- Never
- 1 time
- 2 times
- 3 times or more

22. When was the last time you were trained/ taken a session for CPR

- Never
- Less than one year ago
- More than one year ago, but within 5 years More than 5 years ago

23. Imagine a person collapsed and not breathing normally. Would you be willing to perform CPR?

- Definitely Yes
- Probably Yes
- Probably Not
- Definitely Not

24. what could be the reason/reasons for you to not perform CPR (you can choose more than one option). Please write if you have any other reasons.

- Don’t have the skill to give CPR
- Do not have the confidence to give CPR
- I am not physically fit to perform CPR
- I do not know the person
- Not sure if they need CPR
- Fear I may catch a disease
- Visible signs of vomit/blood
- Smell of alcohol
- I do not want to give mouth-to-mouth resuscitation
- Fear of self-harm
- Fear of being sued/ legal consequences
- Fear about causing injury / making things worse
- Other (please specify)

25. Please indicate your likelihood of performing CPR based on the victim's characteristics:

| Child | Less likely More Likely |
| --- | --- |
| Young Male | Less likely More Likely |
| Elderly (above 60) | Less likely More Likely |
| Young Female | Less likely More Likely |
| Family Memeber | Less likely More Likely |

26. Imagine if a person falls in front of you and suffers a cardiac arrest. What factors would increase the likelihood of you performing a CPR.? You can pick more than one option."

- If I am rewarded for attempting CPR
- If I am sure I am legally protected for attempting CPR (Good Samaritan Law)
- Chest-compressions-only CPR is good enough (no mouth-to-mouth breathing)
- If the gender of the victim is the same as mine
- If the emergency response team guides me on the phone through the process of performing CPR (dispatcher-assisted CPR)
- Other (please specify)

**AED Related**

The next few questions are related to Automated External Defibrillator (AED). Please select the option that best reflects your response to the statements below.
An AED is a portable device that can potentially save the life of someone having a cardiac arrest. It checks the heart's rhythm and sends a shock to the heart to restore a normal rhythm. It is easy-to-use and can guide anyone to use it through simple voice command.

27. Do you feel confident in your ability to use an AED?

- Very confident
- Confident
- Somewhat Confident
- Not Confident

28. Have you ever been trained/taken sessions for using an AED?

- Yes
- No

29. How many times have you been trained/taken a session for using an AED?

- Never
- 1 time
- 2 times
- 3 times or more

30. When was the last time you were trained/taken a session for using an AED?

- Never
- Less than one year ago
- More than one year ago but within 5 years More than 5 years ago

31. Imagine a person collapsed and not breathing normally; would you be willing to use an AED?

- Definitely yes
- Probably yes
- Probably Not
- Definitely Not

32. What could be the reason/reasons for you to not use an AED (you can choose more than one option). Please write if you have any other reasons:

- Don't have the skill to use an AED
- Do not have the confidence to use an AED
- I do not know the person
- Not sure if they need AED intervention
- Fear I may catch a disease
- Visible signs of vomit/blood
- Smell of alcohol
- Fear of self-harm
- Fear of being sued/legal consequences
- Fear about causing injury/making things worse
- Other (please specify)

33. Please indicate your likelihood of using an AED based on the victim's characteristics:

| Child | Less likely More Likely |
| --- | --- |
| Young Male | Less likely More Likely |
| Elderly (above 60) | Less likely More Likely |
| Young Female | Less likely More Likely |
| Family Memeber | Less likely More Likely |

34. Imagine if a person falls in front of you and suffers a cardiac arrest. What factors would increase the likelihood of you using an AED on a stranger? You can pick more than one option."

- If I am rewarded for attempting to use an AED
- If I am sure I am legally protected for attempting to use an AED (Good Samaritan Law)
- If the gender of the victim is the same as mine
- If the emergency response team guides me on the phone through the process of using an AED (dispatcher- assisted AED)
- Other (please specify)

35.Have you witnessed an out-of-hospital cardiac arrest before?

- Yes
- No

## Arabic Version

**التركيبة السكانية**

ما هو عمرك؟

ما هو جنسك؟
ذكر — أنثى

ما هي حالتك الاجتماعية؟
متزوج/ة — أعزب/ة — مطلق/ة أو أرمل/ة

أين تسكن ؟
أبوظبي — العين — دبي — الشارقة — عجمان — أم القيوين — رأس الخيمة — الفجيرة

ما هي جنسيتك؟
إماراتي — عربي — جنوب آسيوي (مثل: الهند، باكستان وبنغلاديش) — جنوب شرق آسيوي (مثل: الفلبين وإندونيسيا) — أوروبي — أفريقي — أخرى (يرجى التحديد)

ما هو مستواك التعليمي؟
ابتدائي/ ثانوي — كلية / جامعة (درجة البكالوريوس أو ما يعادلها) — دراسات عليا (درجة الماجستير أو ما يعادلها) — دراسات عليا متقدمة (دكتوراه في الفلسفة، دكتوراه في الطب، إلخ) — أخرى (يرجى التحديد)

هل أنت مقدم رعاية صحية (طبيب/ممرض/طبيب أسنان/مساعد طبي)؟
نعم — لا

ما هي وظيفتك الحالية؟ الرجاء اختيار واحدة من الفئات التالية:
عامل في مجال الرعاية الصحية — معلم/مدرس/أستاذ — العلم والأبحاث — مهندس تكنولوجيا المعلومات/برمجيات — الموظفون الإداريون — القانون وإنفاذ القانون — العقارات وإدارة الممتلكات — الموارد البشرية — المالية والمحاسبة — الخدمات الاجتماعية/غير الربحية — البناء/العمل اليدوي — التجزئة/المبيعات أو التسويق — الضيافة/صناعة الخدمات — طالب — عاطل عن العمل — أخرى (يرجى التحديد)

ما نوع مكان العمل الذي تعمل فيه؟ الرجاء اختيار واحدة من الفئات التالية:
منشأة رعاية صحية (مثل المستشفى، العيادة) — مدرسة/مؤسسة تعليمية — مكتب/شركات — مختبر — موقع بناء — موقع صناعي/تصنيعي — مكان ضيافة (مثل الفندق، المطعم) — مركز نقل (مثل المطار، محطة القطار) — متجر تجزئة — مطعم/مقهى — منظمة غير ربحية — العمل عن بُعد — إنفاذ القانون / القوات الأمنية — المنزل

ما هو دخلك الشهري؟
عاطل عن العمل — أقل من 4999 درهم — 5000-9999 درهم — 10,000-14,999 درهم — 15,000-19,999 درهم — 20,000-24,999 درهم — 25,000-29,999 درهم — 30,000-34,999 درهم — 36,000-39,999 درهم — 40,000-44,999 درهم — 45,000-49,999 درهم — أكثر من 50,000 درهم

**التاريخ الشخصي والعائلي**

هل تعاني من أمراض القلب؟ (الذبحة الصدرية، احتشاء عضلة القلب، الجلطة، مشاكل قلبية أخرى، أو أجريت عملية في القلب أو أي إجراء آخر في القلب)
نعم — لا

هل يعاني أي فرد من أفراد أسرتك من مرض القلب؟ (الذبحة الصدرية، احتشاء عضلة القلب، الجلطة، مشاكل قلبية أخرى، أو أجرى عملية في القلب أو أي إجراء آخر في القلب)
نعم — لا

هل لديك كبير سن في العائلة أكبر من 65 سنة ويعيش معك؟
نعم — لا

**السكتة القلبية**

هل تشعر بالثقة في قدرتك على التعرف إذا كان شخص ما يعاني من سكتة قلبية؟
نعم — لا

*15.هل تشعر بالثقة في قدرتك على التعرف إذا كان شخص ما يعاني من نوبة قلبية؟

نعم

لا

إذا كنت تشك أن شخصًا ما يعاني من سكتة قلبية، ما هو الرقم الطارئ الصحيح الذي يجب الاتصال به لطلب المساعدة الطبية في الإمارات العربية المتحدة؟
911
999
112
998

**السلوك**

**الإنعاش القلبي الرئوي هو إجراء طارئ يتم إجراؤه لشخص يعاني من سكتة قلبية. يتضمن الضغط على الصدر للحفاظ على سريان الدورة الدموية حتى وصول الإسعاف، يمكن أدائه مع أو بدون التنفس الإنقاذي (أي الإنعاش بإعطاء التنفس من الفم إلى الفم**)

 هل تعتقد أن الإنعاش القلبي الرئوي الذي يقدمه أحد المارة حتى وصول خدمات الطوارئ الطبية يمكن أن يزيد من فرص بقاء الضحية على قيد الحياة؟
نعم
لا

 هل تعتقد أنه يجب تعليم عامة أفراد المجتمع المهارات اللازمة لإجراء الإنعاش القلبي الرئوي؟
نعم
لا

**الإنعاش القلبي الرئوي**

**الأسئلة التالية تتعلق بالإنعاش القلبي الرئوي. يرجى تحديد الاختيار الذي يعكس وجهة نظرك في العبارات أدناهالإنعاش القلبي الرئوي هو إجراء طارئ يتم إجراؤه لشخص يعاني من سكتة قلبية. يتضمن الضغط على الصدر للحفاظ على سريان الدورة الدموية حتى وصول الإسعاف، يمكن أدائه مع أو بدون التنفس الإنقاذي (أي الإنعاش بإعطاء التنفس من الفم إلى الفم**

هل تثق في قدرتك على إجراء الإنعاش القلبي الرئوي؟
واثق جدًا — واثق — واثق إلى حد ما — غير واثق

هل سبق لك أن تدربت/حضرت دورات في الإنعاش القلبي الرئوي؟
نعم — لا

كم عدد المرات التي تدربت فيها/حضرت دورات في الإنعاش القلبي الرئوي؟
لم أتلقى أي تدريب — مرة واحدة — مرتين — ثلاث مرات أو أكثر

متى كانت آخر مرة تدربت فيها/ حضرت دورات في الإنعاش القلبي الرئوي
لم أتلقى أي تدريب — منذ أقل من عام واحد — منذ أكثر من عام واحد، ولكن خلال ال 5 سنوات — أكثر من 5 سنوات

في حالة أنك واجهت أمامك شخص فاقد الوعي ولا يتنفس بشكل طبيعي. هل ستكون مستعداً لإجراء الإنعاش القلبي الرئوي؟
بالتأكيد نعم — ربما نعم — ربما لا — بالتأكيد لا

ما هي الأسباب التي قد تمنعك من إجراء الإنعاش القلبي الرئوي؟ (يمكنك اختيار أكثر من خيار. يرجى الكتابة إذا كان لديك أسباب أخرى.)
ليس لدي المهارة اللازمة لإجراء الإنعاش القلبي الرئوي — ليس لديك الثقة اللازمة لإجراء الإنعاش القلبي الرئوي — أنا لست لائقًا بدنيًا لإجراء الإنعاش القلبي الرئوي — لا أعرف الشخص — لست متأكدًا إذا كان الشخص بحاجة إلى الإنعاش القلبي الرئوي — الخوف من الإصابة بمرض ما — علامات واضحة للقيء / الدم — رائحة الكحول — لا أريد إجراء الإنعاش باستخدام التنفس من الفم إلى الفم — الخوف من إيذاء النفس — الخوف من المقاضاة / المساءلة القانونية — الخوف من التسبب في إصابة / جعل الأمور أكثر سوءاً — أخرى (يرجى التحديد)

.يرجى الإشارة إلى احتمالية إجراء الإنعاش القلبي الرئوي بناءً على خصائص الضحية:

طفل — أقل احتمالًا — أكثر احتمال
فتى شاب — أقل احتمالًا — أكثر احتمالًا
فتاة شابة — أقل احتمالًا — أكثر احتمالًا
كبير سن (فوق 60 سنة) — أقل احتمالًا — أكثر احتمالًا
فرد من أفراد العائلة / صديق العائلة — أقل احتمالًا — أكثر احتمالًا

تخيل لو سقط شخص أمامك وكان يعاني من السكتة القلبية. ما هي العوامل التي من شأنها أن تزيد من احتمالية إجراءك للإنعاش القلبي الرئوي؟ يمكنك اختيار أكثر من اختيار

إذا تمت مكافأتي على محاولتي للإنعاش القلبي الرئوي — إذا كنت متأكدًا من أنني محمي من المساءلة القانونية لإجراء الإنعاش القلبي الرئوي (قانون التطوع لمساعدة الغريب) — الضغط على الصدر في الإنعاش القلبي الرئوي يكفي (بدون إعطاء التنفس من الفم إلى الفم) — إذا كان جنس الضحية هو نفس جنسي — إذا أرشدني فريق الطوارئ عبر الهاتف عن خطوات عمل الإنعاش القلبي الرئوي (الإنعاش القلبي الرئوي بمساعدة مسؤول الاستجابة) — أخرى (يرجى التحديد)

**جهاز إنعاش القلب الخارجي الآلي**

جهاز إنعاش القلب الخارجي الآلي هو جهاز إنعاش آلي يستخدم في حالة توقف القلب ، استخدامه يزيد من احتمالية البقاء على قيد الحياة. حيث يقوم بتحليل دقات القلب وتوجيه صدمة كهربائية لاستعادة دقات القلب الطبيعية. الجهاز سهل الاستخدام ويمكنه إرشاد أي شخص أثناء الاستخدام من خلال توجيه صوتي بسيط.الأسئلة القليلة التالية تتعلق بجهاز إنعاش القلب الخارجي الآلي. يرجى تحديد الاختيار الذي يعكس وجهة نظرك في العبارات أدناه.

هل تشعر بالثقة في قدرتك على استخدام جهاز إنعاش القلب الخارجي الآلي؟

واثق جدًا — واثق — واثق إلى حد ما — غير واثق

هل سبق لك أن تدربت/حضرت دورات في استخدام جهاز إنعاش القلب الخارجي الآلي؟

نعم — لا

كم عدد المرات التي تدربت فيها/حضرت دورات في استخدام جهاز إنعاش القلب الخارجي الآلي؟

لم أتلق أي تدريب — مرة واحدة — مرتين — ثلاث مرات أو أكثر

متى كانت آخر مرة تدربت فيها/حضرت دورات في استخدام جهاز إنعاش القلب الخارجي الآلي؟

لم أتلق أي تدريب — منذ أقل من عام واحد — منذ أكثر من عام، ولكن خلال الـ 5 سنوات — منذ أكثر من 5 سنوات

*35.في حالة أنك واجهت أمامك شخص فاقد الوعي ولا يتنفس بشكل طبيعي؛ هل ستكون مستعداً لاستخدام جهاز إنعاش القلب الخارجي الآلي؟

بالتأكيد نعم

ربما نعم

ربما لا

بالتأكيد لا

*35.في حالة أنك واجهت أمامك شخص فاقد الوعي ولا يتنفس بشكل طبيعي؛ هل ستكون مستعداً لاستخدام جهاز إنعاش القلب الخارجي الآلي؟

بالتأكيد نعم

ربما نعم

ربما لا

بالتأكيد لا

ما هي الأسباب التي قد تمنعك من استخدام جهاز إنعاش القلب الخارجي الآلي؟ (يمكنك اختيار أكثر من خيار. يرجى الكتابة إذا كان لديك أسباب أخرى)

ليس لديك المهارة اللازمة لاستخدام جهاز إنعاش القلب الخارجي الآلي — ليس لديك الثقة اللازمة لاستخدام جهاز إنعاش القلب الخارجي الآلي — لا أعرف الشخص — لست متأكدًا إذا كان الشخص بحاجة إلى جهاز إنعاش القلب الخارجي الآلي — الخوف من الإصابة بمرض ما — علامات واضحة للقيء / الدم — رائحة الكحول — الخوف من إيذاء النفس — الخوف من المقاضاة / المساءلة القانونية — الخوف من التسبب في إصابة / جعل الأمور أكثر سوءاً — أخرى (يرجى التحديد)

.يرجى الإشارة إلى احتمالية استخدام جهاز إنعاش القلب الخارجي الآلي بناءً على خصائص الضحية:

طفل — أقل احتمالًا — أكثر احتمال

فتى شاب — أقل احتمالًا — أكثر احتمالًا

فتاة شابة — أقل احتمالًا — أكثر احتمالًا

كبير سن (فوق 60 سنة) — أقل احتمالًا — أكثر احتمالًا

فرد من أفراد العائلة / صديق العائلة — أقل احتمالًا — أكثر احتمالًا

تخيل لو سقط شخص أمامك وكان يعاني من السكتة القلبية. ما هي العوامل التي من شأنها أن تزيد من احتمالية استخدامك لجهاز إنعاش القلب الخارجي الآلي؟ يمكنك اختيار أكثر من اختيار

إذا تمت مكافأتي على محاولتي استخدام جهاز إنعاش القلب الخارجي الآلي — إذا كنت متأكدًا من أنني محمي من المساءلة القانونية لاستخدام جهاز إنعاش القلب الخارجي الآلي (قانون التطوع لمساعدة الغريب) — إذا كان جنس الضحية هو نفس جنسي — إذا أرشدني فريق الطوارئ عبر الهاتف عن خطوات استخدام جهاز إنعاش القلب الخارجي الآلي (استخدام جهاز إنعاش القلب الخارجي الآلي بمساعدة المسؤول الطبي) — أخرى (يرجى التحديد)

هل شاهدت سكتة قلبية خارج المستشفى من قبل؟

نعم — لا

## Urdu Version

ﮨﻢ ﯾہ ﺗﺤﻘﯿﻖ ﮨﺴﭙﺘﺎل ﮐﮯ ﺑﺎﮨﺮ ﮨﻮﻧﮯ واﻟﮯ ﮐﺎرڈﯾﮏ ارﯾﺴﭧ )دل ﺑﻨﺪ ﮨﻮﻧﺎ( ﮐﮯ واﻗﻌﺎت ﮐﮯ دوران ﻣﻮﻗﻊ ﭘﺮ ﻣﻮﺟﻮد اﻓﺮاد ﮐﮯ ردﻋﻤﻞ ﻣﯿﮟ رﮐﺎوﭨﻮں، اور ﻣﻌﺎوﻧﯿﻦ ﮐﮯ ﺑﺎرے ﻣﯿﮟ ﻣﻌﻠﻮﻣﺎت ﺣﺎﺻﻞ ﮐﺮﻧﮯ ﮐﮯ ﻟﯿﮯ ﮐﺮ رﮨﮯ ﮨﯿﮟ۔ ان ﻋﻮاﻣﻞ ﮐﻮ ﺳﻤﺠﮭﻨﺎ

ان واﻗﻌﺎت ﻣﯿﮟ ﺑﻘﺎ ﮐﮯ اﻣﮑﺎﻧﺎت ﮐﻮ ﺑﮩﺘﺮ ﺑﻨﺎﻧﮯ ﮐﮯ ﻟﯿﮯ ﺑﮯ ﺣﺪ ﺿﺮوری ﮨﮯﻓﻮاﺋﺪ: اﮔﺮﭼہ آپ ﮐﻮ ﺑﻄﻮر ﺷﺮﮐﺎء ﮐﻮﺋﯽ ﺑﺮاه راﺳﺖ ﻓﺎﺋﺪه ﻧﮩﯿﮟ ﮨﮯ، اس ﺗﺤﻘﯿﻖ ﻣﯿﮟ آپ ﮐﺎ ﺗﻌﺎون ﻋﻠﻢ ﮐﮯ ﻓﺮوغ ﻣﯿﮟ ﻣﺪد ﮐﺮے ﮔﺎ۔ اﭘﻨﯽ ﺑﺼﯿﺮت اور ﺗﺠﺮﺑﺎت ﮐﻮ ﺷﯿﺌﺮ

ﮐﺮﮐﮯ، آپ ﻣﺴﺘﻘﺒﻞ ﻣﯿﮟ ﺟﺎﻧﯿﮟ ﺑﭽﺎﻧﮯ ﻣﯿﮟ ﻣﺪدﮔﺎر ﮨﻮں ﮔﮯ۔ﺧﻄﺮه: اس ﻣﻄﺎﻟﻌﮯ ﻣﯿﮟ ﺣﺼہ ﻟﯿﻨﮯ ﺳﮯ ﮐﻮﺋﯽ ﻣﺘﻮﻗﻊ ﺧﻄﺮات واﺑﺴﺘہ ﻧﮩﯿﮟ ﮨﯿﮟ۔رازداری: اس ﺳﺮوے ﻣﯿﮟ آپ ﮐﯽ طﺮف ﺳﮯ ﻓﺮاﮨﻢ ﮐﺮده ﮐﺴﯽ ﺑﮭﯽ ﻣﻌﻠﻮﻣﺎت ﮐﻮ اﻧﺘﮩﺎﺋﯽ رازداری اور اﺣﺘﯿﺎط ﺳﮯ رﮐﮭﺎ ﺟﺎﺋﮯ ﮔﺎ۔ آپ ﮐﮯ ﺟﻮاﺑﺎت ﮐﻮ ﻣﺤﻔﻮظ طﺮﯾﻘﮯ ﺳﮯ رﮐﮭﺎﺟﺎﺋﮯ ﮔﺎ اور ﺻﺮف ﻣﺠﺎز ﺗﺤﻘﯿﻘﺎﺗﯽ ﭨﯿﻢ ﮐﮯ اراﮐﯿﻦ ﺗﮏ رﺳﺎﺋﯽ ﮨﻮﮔﯽ۔ آپ ﮐﯽ ﺷﻨﺎﺧﺖ ﮔﻤﻨﺎم رﮨﮯ ﮔﯽ، ﯾﻌﻨﯽ آپ ﮐﮯ ﺟﻮاﺑﺎت ﮐﮯ ﺳﺎﺗﮭ ﮐﻮﺋﯽ ذاﺗﯽ ﺷﻨﺎﺧﺘﯽ ﻣﻌﻠﻮﻣﺎت

ﻣﻨﺴﻠﮏ ﻧﮩﯿﮟ ﮨﻮں ﮔﯽ۔ﺷﻤﻮﻟﯿﺘﺎس ﻣﻄﺎﻟﻌﮯ ﻣﯿﮟ آپ ﮐﯽ ﺷﻤﻮﻟﯿﺖ ﻣﮑﻤﻞ طﻮر ﭘﺮ رﺿﺎﮐﺎراﻧہ ﮨﮯ۔ آپ ﮐﻮ ﺣﺼہ ﻟﯿﻨﮯ ﮐﯽ ﮐﻮﺋﯿﻤﺠﺒﻮری ﯾﺎ زﺑﺮدﺳﺘﯽ ﻧﮩﯿﮟ ﮨﮯاور اﮔﺮ آپ ﺣﺼہ ﻧہ ﻟﯿﻨﮯ ﯾﺎ ﮐﺴﯽ ﺑﮭﯽ وﻗﺖ ﻣﻄﺎﻟﻌﮯ ﺳﮯ دﺳﺘﺒﺮدار ﮨﻮﻧﮯ ﮐﺎ ﻓﯿﺼﻠہ

ﮐﺮﺗﮯ ﮨﯿﮟ، ﺗﻮ اس ﮐﮯ ﮐﻮﺋﯽ ﻣﻨﻔﯽ ﻧﺘﺎﺋﺞ ﻧﮩﯿﮟ ﮨﻮں ﮔﮯ۔ ﺣﺼہ ﻟﯿﻨﮯ ﭘﺮ رﺿﺎﻣﻨﺪی ظﺎﮨﺮ ﮐﺮﮐﮯ، آپ اس ﺳﺮوے ﮐﻮ ﻣﮑﻤﻞ ﮐﺮﻧﮯ اور ﮨﻤﺎری ﺗﺤﻘﯿﻖ ﮐﯽ ﮐﻮﺷﺸﻮں ﻣﯿﮟ ﺗﻌﺎون ﮐﺮﻧﮯ ﭘﺮ اﭘﻨﯽ رﺿﺎ ﻣﻨﺪی ﮐﯽ ﻧﺸﺎﻧﺪﮨﯽ ﮐﺮﺗﮯ ﮨﯿﮟ۔ اﮔﺮ آپ ﮐﮯ ﻣﻄﺎﻟﻌﮯ

ﮐﮯ ﺑﺎرے ﻣﯿﮟ ﮐﻮﺋﯽ ﺳﻮاﻻت ﯾﺎ ﺧﺪﺷﺎت ﮨﯿﮟ، ﺗﻮ ﺑﺮاه ﮐﺮم ﮨﻢ ﺳﮯ راﺑﻄہ ﮐﺮﯾﮟ۔ اس اﮨﻢ ﺗﺤﻘﯿﻘﯽ ﮐﻮﺷﺶ ﻣﯿﮟ ﺣﺼہ ﻟﯿﻨﮯ ﭘﺮ

mfarooq@uaeu.ac.ae :ﻏﻮر ﮐﺮﻧﮯ ﮐﮯ ﻟﯿﮯ آپ ﮐﺎ ﺷﮑﺮﯾہ۔راﺑﻄہ

آپ ﮐﯽ ﻋﻤﺮ ﮐﯿﺎ ﮨﮯ؟

آپ ﮐﯽ ﺟﻨﺲ ﮐﯿﺎ ﮨﮯ؟. *

ﻣﺮد ﻋﻮرت

آپ ﮐﯽ ازدواﺟﯽ ﺣﯿﺜﯿﺖ ﮐﯿﺎ ﮨﮯ؟. *

ﺷﺎدی ﺷﺪه ﻏﯿﺮ ﺷﺎدی ﺷﺪه

طﻼق ﯾﺎﻓﺘہ/ﺑﯿﻮه

آپ ﮐﮩﺎں ﻣﻘﯿﻢ ﮨﯿﮟ. *

اﺑﻮظﮩﺒﯽ

اﻟﻌﯿﻦ

دﺑﺌﯽ

ﺷﺎرﺟہ

ﻋﺠﻤﺎن

ام اﻟﻘﻮﯾﻦ

راس اﻟﺨﯿﻤہ

ﻓﺠﯿﺮه

آپ ﮐﺎ ﺗﻌﻠﻖ ﮐﮩﺎں ﺳﮯ ﮨﮯ. *

اﻣﺎراﺗﯽ دﯾﮕﺮ ﻋﺮب

ﺟﻨﻮﺑﯽ اﯾﺸﯿﺎﺋﯽ )ﺟﯿﺴﮯ، ﺑﮭﺎرﺗﯽ، ﭘﺎﮐﺴﺘﺎﻧﯽ، ﺑﻨﮕﻠہ دﯾﺸﯽ

ﻣﺸﺮﻗﯽ اﯾﺸﯿﺎﺋﯽ )ﺟﯿﺴﮯ، ﻓﻠﭙﺎﺋﻨﯽ، اﻧﮉوﻧﯿﺸﯿﺎﺋﯽ

ﻣﻐﺮﺑﯽ اﻓﺮﯾﻘﯽ

دﯾﮕﺮ )ﺑﺮاه ﮐﺮم وﺿﺎﺣﺖ ﮐﺮﯾﮟ

آپ ﮐﯽ ﺗﻌﻠﯿﻤﯽ ﺳﻄﺢ ﮐﯿﺎ ﮨﮯ؟. *

اﺑﺘﺪاﺋﯽ/ﺛﺎﻧﻮی اﺳﮑﻮل

ﮐﺎﻟﺞ/ﯾﻮﻧﯿﻮرﺳﭩﯽ )ﺑﯿﭽﻠﺮز ڈﮔﺮی ﯾﺎ اس ﮐﮯ ﻣﺴﺎوی(

ﭘﻮﺳﭧ ﮔﺮﯾﺠﻮﯾﭧ اﺳﮑﻮل )ﻣﺎﺳﭩﺮز ڈﮔﺮی ﯾﺎ اس ﮐﮯ ﻣﺴﺎوی

اﻋﻠٰﯽ ﭘﻮﺳﭧ ﮔﺮﯾﺠﻮﯾﭧ اﺳﮑﻮل )ﭘﯽ اﯾﭻ ڈی، اﯾﻢ ڈی وﻏﯿﺮ

دﯾﮕﺮ )ﺑﺮاه ﮐﺮم وﺿﺎﺣﺖ ﮐﺮﯾﮟ

ﮐﯿﺎ آپ ﺻﺤﺖ /ﮨﺴﭙﺘﺎل ﮐﮯ ﺷﻌﺒﮯ ﺳﮯ ﻣﻨﺴﻠﮏ ﮨﯿﮟ؟. *

ﮨﺎں ﻧﮩﯿﮟ

:آپ ﮐﺎ ﻣﻮﺟﻮده ﭘﯿﺸہ ﮐﯿﺎ ﮨﮯ؟ ﺑﺮاه ﮐﺮم درج ذﯾﻞ ﻣﯿﮟ ﺳﮯ اﯾﮏ ﮐﺎ اﻧﺘﺨﺎب ﮐﺮﯾﮟ. *

ﺻﺤﺖ/ﮨﺴﭙﺘﺎل ﺳﮯ واﺑﺴﺘہ اﺳﺘﺎد/ﺗﻌﻠﯿﻤﯽ ﻣﺎﮨﺮ

ﺳﺎﺋﻨﺲ اور ﺗﺤﻘﯿﻖ

آﺋﯽ ﭨﯽ/ﺳﺎﻓﭧ وﯾﺌﺮ اﻧﺠﯿﻨﺌﺮ

اﻧﺘﻈﺎﻣﯽ ﻋﻤﻠہ

ﻗﺎﻧﻮﻧﯽ اور ﻗﺎﻧﻮن ﻧﺎﻓﺬ ﮐﺮﻧﮯ واﻟﮯ ادارے

رﺋﯿﻞ اﺳﭩﯿﭧ اور ﭘﺮاﭘﺮﭨﯽ ﻣﯿﻨﺠﻤﻨﭧ

اﻧﺴﺎﻧﯽ وﺳﺎﺋﻞ، اﻓﺮادی ﻗﻮت ﯾﺎ وﺳﺎﺋﻞ

ﻣﺎﻟﯿﺎت اور اﮐﺎؤﻧﭩﻨﮓ

ﻓﻼﺣﯽ اداره

ﺗﻌﻤﯿﺮات/دﺳﺘﯽ ﻣﺤﻨﺖ

ﺧﻮرده/ﻓﺮوﺧﺖ ﯾﺎ ﻣﺎرﮐﯿﭩﻨﮓ

ﮨﻮﭨﻠﻨﮓ/ﺳﺮوس اﻧﮉﺳﭩﺮی

طﺎﻟﺐ ﻋﻠﻢ

ﺑﮯ روزﮔﺎر

دﯾﮕﺮ )ﺑﺮاه ﮐﺮم وﺿﺎﺣﺖ ﮐﺮﯾﮟ

آپ کس قسم کی ورک پلیس میں کام کرتے ہیں؟ براہ کرم درج ذیل زمروں میں سے ایک کا انتخاب کریں*

صحت کی سہولت (جیسے اسپتال، کلینک)

تعلیمی ادارہ (جیسے اسکول، کالج، یونیورسٹی)

دفتر یا کارپوریٹ ماحول

لیبارٹری

تعمیراتی سائٹ

صنعتی یا مینوفیکچرنگ سائٹ

ہوٹل یا مہمان نوازی کی جگہ

ٹرانسپورٹ کا مرکز (جیسے ہوائی اڈہ، ریلوے اسٹیشن)

خوردہ دکان

ریسٹورنٹ یا کیفے

فلاحی ادارہ

گھر سے یا آن لائن کام

قانون نافذ کرنے والے ادارے یا سیکیورٹی فورسز

گھریلو خاتون یا گھر پر موجود

بے روزگار

دیگر (براہ کرم وضاحت کریں)

آپ ﮐﯽ ﻣﺎﮨﺎﻧہ آﻣﺪﻧﯽ ﮐﯿﺎ ﮨﮯ. *

ﺑﮯ روزﮔﺎر

<4999 AED

5000-9999 AED

10,000- 14,999 AED

15,000-19,999 AED

20,000- 24,999 AED

25,000-29,999 AED

30,000-34,999 AED

36,000 - 39,999 AED

40,000-44,999 AED

40,000-44,999 AED

>50,000

ذاتی اور خاندانی تاریخ

کیا آپ کسی قسم کی دل کی بیماری کا شکار ہیں؟ (دل کا دورہ، فالج، دیگر قلبی عوارض، دل کی سرجری/عمل ہوا ہے*

ہاں / نہیں

کیا آپ کے گھر والوں میں سے کسی کو دل کی بیماری ہے؟ (دل کا دورہ، فالج، دیگر قلبی عوارض، دل کی سرجری/عمل ہوا ہے)

ہاں / نہیں

کیا آپ کے گھر میں کوئی بزرگ ہے جو 65 سال سے زیادہ عمر کا ہے اور آپ کے ساتھ رہائش پذیر ہے؟ *

ہاں / نہیں

کیا آپ کو یقین ہے کہ آپ یہ پہچان سکتے ہیں کہ کوئی شخص دل بند ہو جانے (کارڈیک اریسٹ) کا شکار ہے؟ *

— ہاں / نہیں

کیا آپ کو یقین ہے کہ آپ یہ پہچان سکتے ہیں کہ کسی شخص کو دل کا دورہ پڑ رہا ہے؟ *

— ہاں / نہیں

اگر آپ کو شبہ ہو کہ کوئی شخص دل بند ہو جانے (کارڈیک اریسٹ) کا شکار ہے، تو متحدہ عرب امارات میں طبی مدد کے لیے صحیح ایمرجنسی نمبر کیا ہے؟

911

999

112

998

**خیالات**

کارڈیوپلمونری ریسیسیٹیشن ایک ایمرجنسی طریقۂ کار ہے جو دل بند ہو جانے (کارڈیک اریسٹ) کا شکار شخص پر کیا جاتا ہے۔ اس میں دل کی دھڑکن کو برقرار رکھنے کے لیے سینے پر ہاتھوں کی مدد سے دباؤ (کمپریشنز) دیے جاتے ہیں جب تک کہ ایمبولینس نہ پہنچ جائے۔ یہ منہ سے ریسکیو سانس کے ساتھ یا بغیر ریسکیو سانس کے دیا جا سکتا ہے۔

آپ کے خیال میں موقع پر موجود شخص کی طرف سے دیا گیا کارڈیوپلمونری ریسیسیٹیشن (سی پی آر) متاثرہ شخص* کے بچنے کے امکانات بڑھا سکتا ہے؟ — ہاں / نہیں

آپ کے خیال میں کیا عام لوگوں کو (سی پی آر) کا طریقۂ کار سیکھنا چاہیے؟ — ہاں / نہیں*

**ﺳﯽ ﭘﯽ آر ﺳﮯ ﻣﺘﻌﻠﻖ**

**کارڈیوپلمونری ریسیسیٹیشن ایک ایمرجنسی طریقۂ کار ہے جو دل بند ہو جانے (کارڈیک اریسٹ) کا شکار شخص پر کیا جاتا ہے۔ اس میں دل کی دھڑکن کو برقرار رکھنے کے لیے سینے پر ہاتھوں کی مدد سے دباؤ (کمپریشنز) دیے جاتے ہیں جب تک کہ ایمبولینس نہ پہنچ جائے۔ یہ منہ سے ریسکیو سانس کے ساتھ یا بغیر ریسکیو سانس کے دیا جا سکتا ہے۔**

کیا آپ کو اپنے سی پی آر کرنے کی صلاحیت پر اعتماد ہے؟ *

ﻣﮑﻤﻞ اﻋﺘﻤﺎد ﮨﮯ

اعتماد ہے

کچھ حد تک اعتماد ہے

اعتماد نہیں ہے

آپ نے کبھی سی پی آر کے لیے تربیت حاصل کی ہے یا سیشن لیے ہیں؟ *

— ہاں / نہیں

آپ نے کتنی بار سی پی آر کی تربیت حاصل کی ہے یا سیشن لیے ہیں؟ *

کبھی نہیں

ایک بار

دو بار

تین بار یا زیادہ

آخری بار آپ نے سی پی آر کی تربیت کب حاصل کی تھی یا سیشن لیا تھا؟ *

کبھی نہیں

ایک سال سے کم عرصہ پہلے

ایک سال سے زیادہ عرصہ پہلے، مگر پانچ سال کے اندر

پانچ سال سے زیادہ عرصہ پہلے

تصور کریں کہ کوئی شخص بے ہوش ہو گیا ہے اور معمول کے مطابق سانس نہیں لے رہا۔ کیا آپ سی پی آر کرنے کے لیے تیار ہوں گے؟

یقینی طور پر ہاں

شاید ہاں

شاید نہیں

یقینی طور پر نہیں

آپ کے سی پی آر نہ کرنے کی ان میں سے کیا وجوہات ہو سکتی ہیں؟ آپ ایک سے زیادہ وجوہات کا انتخاب کر سکتے ہیں۔ اگر کوئی اور وجہ ہے تو براہ کرم لکھ کر جواب دیں۔

سی پی آر دینے کی مہارت نہیں ہے

سی پی آر دینے کی صلاحیت پر اعتماد نہیں ہے

جسمانی طور پر سی پی آر دینے کے لیے فٹ نہیں ہوں

میں شخص کو نہیں جانتا

نہیں معلوم کہ انہیں سی پی آر کی ضرورت ہے

بیماری لگنے کا خوف

الٹی یا خون کی واضح علامات

شراب کی بو

منہ سے منہ ریسیسیٹیشن دینے کا ارادہ نہیں

خود کو نقصان پہنچانے کا خوف

مقدمے یا قانونی نتائج کا خوف

چوٹ لگانے یا حالت بگاڑنے کا خوف

دیگر (براہ کرم وضاحت کریں)

براہ کرم متاثرہ شخص کی خصوصیات کے مطابق اپنے سی پی آر کرنے کے امکانات کی وضاحت کریں۔

بچہ / بچی — کم ممکن ہے — زیادہ ممکن ہے
جوان مرد — کم ممکن ہے — زیادہ ممکن ہے
جوان عورت — کم ممکن ہے — زیادہ ممکن ہے
بزرگ (60 سال سے زیادہ) — کم ممکن ہے — زیادہ ممکن ہے
خاندان کا رکن / دوست — کم ممکن ہے — زیادہ ممکن ہے

تصور کریں کہ اگر کوئی شخص آپ کے سامنے بے ہوش ہو جائے اور دل بند ہونے کا شکار ہو جائے تو آپ کے سی پی آر کرنے کے امکانات کو کون سے عوامل بڑھا سکتے ہیں؟ آپ ایک سے زیادہ اختیار منتخب کر سکتے ہیں:

اگر مجھے سی پی آر کرنے کی کوشش پر انعام ملے

اگر مجھے یقین ہو کہ سی پی آر کرنے کے لیے مجھے قانونی تحفظ حاصل ہے (اچھا سامری قانون)

سینے کی کمپریشنز پر مشتمل سی پی آر کافی ہو (منہ سے منہ سانس دینے کی ضرورت نہ ہو)

اگر متاثرہ شخص کا جنس میرے جیسا ہو

اگر ایمرجنسی رسپانس ٹیم مجھے فون پر سی پی آر کرنے کی رہنمائی کرے (ڈسپیچر-اسسٹڈ سی پی آر)

دیگر (براہ کرم وضاحت کریں

اے ای ڈی سے متعلق

**اگلے چند سوالات آٹومیٹڈ ایکسٹرنل ڈیفبریلیٹر (اے ای ڈی) کے متعلق ہیں۔ براہ کرم نیچے دیے گئے بیانات پر اپنے جوابات کا بہترین انتخاب کریں۔
اے ای ڈی ایک پورٹیبل ڈیوائس (جو ہلکا پھلکا ہو اور کہیں بھی لے جایا جا سکے) ہے جو دل بند ہو جانے کی صورت میں کسی شخص کی جان بچا سکتی ہے۔ یہ دل کی دھڑکن کی جانچ کرتی ہے اور دل کو جھٹکا دے کر معمول کی دھڑکن بحال کرتی ہے۔ اسے استعمال کرنا آسان ہے اور یہ کسی کو بھی سادہ آواز کی ہدایات کے ذریعے استعمال کرنے میں رہنمائی فراہم کر سکتی ہے۔**

کیا آپ کو اپنی آٹومیٹڈ ایکسٹرنل ڈیفبریلیٹر (اے ای ڈی) استعمال کرنے کی صلاحیت پر اعتماد ہے؟

مکمل اعتماد ہے
اعتماد ہے
کچھ حد تک اعتماد ہے
اعتماد نہیں ہے

کیا آپ نے کبھی آٹومیٹڈ ایکسٹرنل ڈیفبریلیٹر (اے ای ڈی) استعمال کرنے کی تربیت حاصل کی ہے؟

ہاں / نہیں

آپ نے کتنی بار آٹومیٹڈ ایکسٹرنل ڈیفبریلیٹر (اے ای ڈی) استعمال کرنے کی تربیت حاصل کی ہے؟

ایک بار
دو بار
تین بار یا اس سے زیادہ

آپ نے آخری بار آٹومیٹڈ ایکسٹرنل ڈیفبریلیٹر (اے ای ڈی) استعمال کرنے کی تربیت کب حاصل کی تھی؟

کبھی نہیں
ایک سال سے کم عرصہ پہلے
ایک سال سے زیادہ مگر پانچ سال کے اندر
پانچ سال سے زیادہ پہلے

تصور کریں کہ ایک شخص آپ کے سامنے بے ہوش ہو جائے اور معمولی طریقے سے سانس نہ لے رہا ہو؛ کیا آپ آٹومیٹڈ ایکسٹرنل ڈیفبریلیٹر (اے ای ڈی) استعمال کرنے کے لیے تیار ہوں گے؟

یقینی طور پر ہاں
شاید ہاں
شاید نہیں
یقینی طور پر نہیں

آپ کے آٹومیٹڈ ایکسٹرنل ڈیفبریلیٹر (اے ای ڈی) استعمال نہ کرنے کی کیا وجوہات ہو سکتی ہیں؟ (آپ ایک سے زیادہ وجوہات منتخب کر سکتے ہیں۔ براہ کرم اگر کوئی اور وجہ ہو تو اسے بھی تحریر کریں۔)

میرے پاس اے ای ڈی استعمال کرنے کی صلاحیت نہیں ہے
اے ای ڈی کے استعمال کی صلاحیت پر اعتماد نہیں ہے
میں شخص کو نہیں جانتا
یقین نہیں ہے کہ انہیں اے ای ڈی کی ضرورت ہے
بیماری لگنے کا خوف
قے/خون کے واضح علامات
شراب کی بو
خود کو نقصان پہنچانے کا خوف
مقدمے یا قانونی نتائج کا خوف
چوٹ پہنچانے یا حالات کو مزید بگاڑنے کا خوف
دیگر (براہ کرم وضاحت کریں)

متاثرہ شخص کے مطابق اپنے آٹومیٹڈ ایکسٹرنل ڈیفبریلیٹر (اے ای ڈی) استعمال کرنے کے امکانات کی وضاحت کریں۔

بچہ / بچی — کم ممکن ہے — زیادہ ممکن ہے
جوان مرد — کم ممکن ہے — زیادہ ممکن ہے
جوان عورت — کم ممکن ہے — زیادہ ممکن ہے
بزرگ (60 سال سے زیادہ) — کم ممکن ہے — زیادہ ممکن ہے
خاندان کا رکن / دوست — کم ممکن ہے — زیادہ ممکن ہے

اگر کوئی شخص آپ کے سامنے بے ہوش ہو جائے اور دل بند ہونے کا شکار ہو جائے تو آپ کے آٹومیٹڈ ایکسٹرنل ڈیفبریلیٹر (اے ای ڈی) استعمال کرنے کے امکانات کو کون سے عوامل بڑھا سکتے ہیں؟ آپ ایک سے زیادہ اختیار منتخب کر سکتے ہیں۔

اگر مجھے آٹومیٹڈ ایکسٹرنل ڈیفبریلیٹر (اے ای ڈی) استعمال کرنے پر انعام دیا جائے:

اگر مجھے یقین ہو کہ آٹومیٹڈ ایکسٹرنل ڈیفبریلیٹر (اے ای ڈی) استعمال کرنے پر قانونی تحفظ حاصل ہے
اگر متاثرہ شخص کا جنس میرے جیسا ہو
ایمرجنسی رسپانس ٹیم مجھے فون پر آٹومیٹڈ ایکسٹرنل ڈیفبریلیٹر (اے ای ڈی) استعمال کرنے کے عمل کی رہنمائی فراہم کرے
دیگر (براہ کرم وضاحت کریں)

کیا آپ نے کسی ہسپتال کے باہر دل بند ہونے کا واقعہ دیکھا ہے؟
ہاں
نہیں
